# NMR Signatures of Transition-Metal Nuclei: From Local Environments and Electronic Structures to Reactivity Descriptors in Molecular and Heterogeneous Catalysis

**DOI:** 10.1021/jacsau.5c00061

**Published:** 2025-07-16

**Authors:** Zachariah J. Berkson, Weicheng Cao, Domenico Gioffrè, Christoph J. Kaul, Lukas Lätsch, Yuya Kakiuchi, Alexander Yakimov, Christophe Copéret

**Affiliations:** 1 Department of Chemistry and Applied Biosciences, 27219 ETH Zürich, Zürich CH-8093, Switzerland; 2 Chemical Engineering, School for the Engineering of Matter, Transport and Energy, 7864 Arizona State University, Tempe, Arizona 85287, United States

**Keywords:** solid-state NMR, NMR signatures, transition metals, DFT calculations, electronic structures, coordination environment, structure−activity relationships

## Abstract

This Perspective summarizes the current state of the art in understanding the local environments of metal sites across homogeneous and heterogeneous catalysts by means of solid-state nuclear magnetic resonance (NMR), augmented with first-principles density functional theory (DFT) calculations, focusing on transition-metal nuclei and emphasizing the potential of this approach for understanding reactivity. We illustrate in particular how NMR parameters of transition-metal nuclei provide unique insights into the electronic structures and coordination environments of metal sites, complementary to information that can be obtained from ^13^C, ^15^N, or ^17^O NMR parameters of metal-bound ligands. Using the examples of solid-state NMR analyses of supported and molecular systems containing NMR-active transition-metal nuclei (^95^Mo, ^195^Pt, ^109^Ag, ^183^W, ^51^V, and ^47/49^Ti), we show how NMR parameters can be determined and related to structural and electronic features of molecular and surface metal sites. Moreover, analyzing the origins of the chemical shift tensors of these metal nuclei through DFT computations helps to connect NMR signatures to specific local coordination environments and electronic structures (frontier molecular orbitals) and the corresponding reactivity of specific metal sites, thereby opening the possibility of establishing structure–activity relationships across catalytic systems, including industrially relevant heterogeneous catalysts.

## Graphical abstract

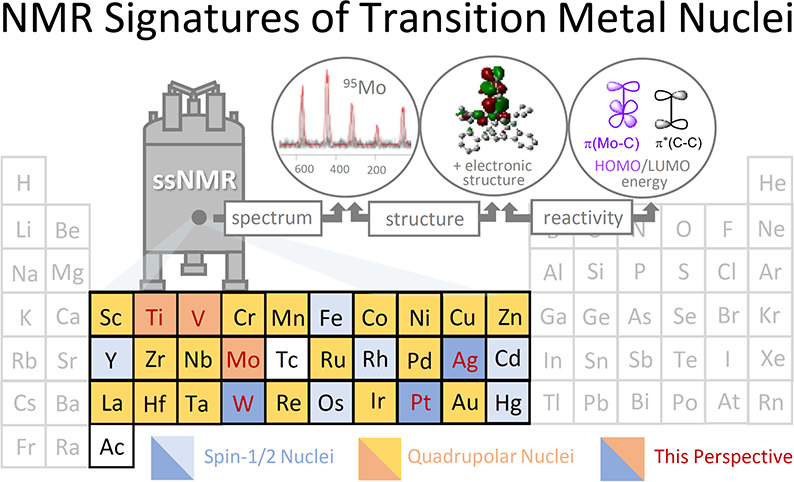

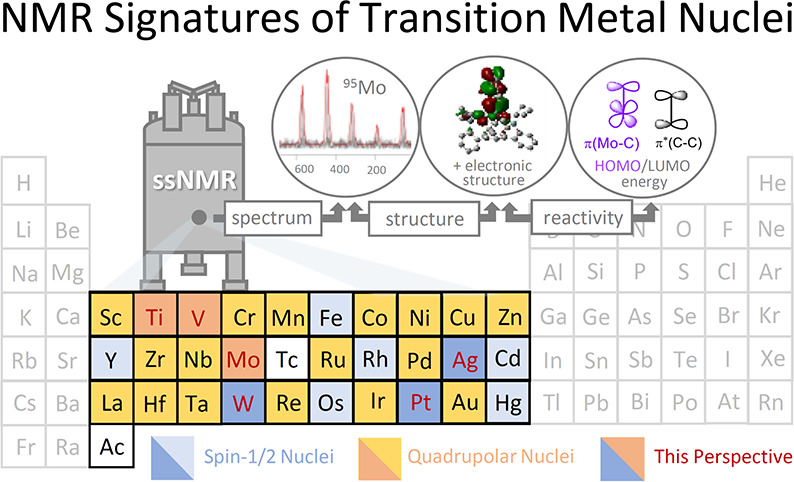

## 1 Introduction

The unique properties of transition metals in homogeneous and heterogeneous catalysts depend on their coordination environments, defined by the nature and geometry of specific metal-bound ligands or atoms, which together determine the electronic structures and reactivity.^1^ Thus, major efforts have been devoted to collecting and interpreting information about the local electronic structures of metal sites in both molecular and heterogeneous catalysts using a range of spectroscopic techniques, such as UV–vis, X-ray-based methods (XPS, UPS, XANES, etc.), electron paramagnetic resonance (EPR), and Mössbauer spectroscopy, all of which provide distinct information related to electronic structures.^2–4^ In this context, nuclear magnetic resonance (NMR) spectroscopy has been much less considered, even if poised to uncover unique information, paralleling EPR spectroscopy for paramagnetic systems.^5,6^ By comparison, NMR spectroscopy has been mostly used as an element-specific tool to identify structural motifs by leveraging the sensitivity of isotropic chemical shifts (δ_*iso*_) to local environments.^7–11^ Multidimensional NMR approaches are particularly powerful for determining 3D structures by establishing through-bond connectivities and/or spatial proximities of nuclei in organic and inorganic compounds and materials, or biological systems.^12–20^

Yet, the NMR response of a given nucleus, including the chemical shift, δ, in a molecule or material also encodes detailed information about the electronic structure at the nuclear site. Chemical shifts arise from the electron distribution around a nucleus and, therefore, depend on its specific orientation with respect to the external magnetic field, *B*_*0*_, specifically in relation to its local electronic structure (e.g., hybridization). The chemical shift tensor (CST) is a diagonalizable second rank tensor where the mean of the principal components, δ_*11*_ ≥ δ_*22*_ ≥ δ_*33*_, corresponds to the isotropic chemical shift typically measured in solution, δ_*iso*_.^21,22^ Due to dynamic averaging, δ_*iso*_ is typically the only parameter measured in solution NMR of solubilized molecules. In contrast, the powder NMR spectrum measured by solid-state NMR of static samples manifests and enables extraction of these principal components, which can be expressed using for instance the Herzfeld–Berger convention:^23^

$$\delta_{iso}=\frac{\left(\delta_{11}+\delta_{22}+\delta_{33}\right)}{3};\Omega =\delta_{11}-\delta_{33};\kappa =\frac{3\left(\delta_{22}-\delta_{iso}\right)}{\Omega }$$

where Ω is referred to as the span and κ is the skew of the chemical shift anisotropy (CSA).

Though δ_*iso*_ contains averaged information, it is a powerful and widely used descriptor for local environment largely thanks to literature databases and empirical rules. By comparison, the individual principal components of the CST directly relate to the symmetry and electronic structure of the observed nuclei. This information is only accessible from solid-state NMR.

For quadrupolar nuclei (*I* > 1/2), the solid-state NMR signatures are more complex because the coupling of the nuclear quadrupole moment with the electric field gradient (EFG) results in further signal broadening. The EFG is represented by a traceless second rank tensor (*V*_*33*_ + *V*_*22*_ + *V*_*11*_ = 0, |*V*_*33*_| ≥ |*V*_*22*_| ≥ |*V*_*11*_|) that is commonly described by two parameters,^24^ the quadrupolar coupling constant (*C*_*Q*_) and the asymmetry parameter (η_Q_):

$$C_{Q}=\frac{eQV_{33}}{h};\eta_{Q}=\frac{V_{11}-V_{22}}{V_{33}}$$

where *e* is the elementary charge, *Q* is the electric quadrupole moment of a nucleus, and *h* is Planck’s constant.

In addition, the relative orientation of the chemical shift and EFG tensors affect the NMR signatures and are described by three Euler angles (α, β, γ). While CSTs are driven mostly by the electronic structure, the nature and relative energy of both virtual and occupied molecular orbitals (*vide infra*), the EFG tensor reflects the local charge distribution around the observed nucleus, hence its sensitivity to the symmetry.^25,26^ Note that the accurate extraction of these parameters (δ_*iso*_, Ω, κ, *C*_*Q*_, η_*Q*_, α, β, γ) typically requires the use of multiple NMR measurements at different fields and/or spinning rates; effective experimental methodologies depend on which interaction dominates (quadrupolar or chemical shift anisotropy). Comprehensive analysis of these parameters requires careful measurement but also computation via quantum mechanical approaches, e.g., density functional theory (DFT). This enables to pinpoint the exact molecular-level origin of these NMR parameters (*vide infra*).

In fact, recent studies have demonstrated the power of NMR chemical shift analysis to interrogate metal–ligand bonding and draw structure–reactivity patterns for organometallic intermediates,^27–37^ focusing mostly on interpreting the NMR signatures of the ligand nuclei directly bound to a metal site. Indeed, these nuclei typically exhibit favorable NMR characteristics: spin 1/2 or with small quadrupolar moments, high gyromagnetic ratio γ (sensitivity), and readily (naturally or synthetically) available in an isotopic pure form. Such nuclei, notably, ^1^H, ^13^C, ^15^N, ^17^O, ^19^F, and ^31^P, enable relatively straightforward measurement and determination of their NMR parameters toward extracting detailed, albeit indirect, information about the metal sites to which the observed nuclei are bound. These analyses are very informative because the NMR signatures of organometallic intermediates display chemical shift windows typically one order of magnitude larger than what is observed for the corresponding organic compounds (hundreds vs tens of ppm for ^13^C).

Considering that almost all transition metals exhibit at least one stable, NMR-active isotope and have even larger chemical shift windows (>100 ppm and up to several 10,000 ppm), one may expect to resolve even more detailed information down to subtle changes in electronic structures. Following our review on chemical analysis of nuclei typical for ligands (e.g., ^13^C, ^15^N, ^31^P),^38^ this Perspective focuses on evaluating the NMR signatures of transition-metal sites across a range of systems from well-defined organometallic molecules to multicomponent heterogeneous catalysts used in industry. The Perspective is divided into five sections based on representative examples of catalytic systems and their particular NMR challenges.

Sections 2 and 3 introduce key experimental and computational protocols for obtaining and theoretical background for interpreting NMR spectroscopic signatures while highlighting the specific challenges related to solid-state NMR analyses of transition-metal nuclei. The following sections focus on case studies from understanding electronic structures of molecular and supported catalysts to solving the structure of surface sites in classical heterogeneous catalysts. Specifically, Section 4 is devoted to NMR analysis of well-defined d^0^ molecular complexes relevant to catalytic olefin and alkyne metathesis reactions, hence focusing on two of the Group 6 transition-metal elements, ^95^Mo and ^183^W, showing how their NMR parameters enable development of powerful structure–activity relationships. This section highlights the challenges, complementarity, and advantages of understanding the NMR signatures of transition-metal nuclei vs those of the nuclei in ligands bound to the metal centers (e.g., ^13^C, ^17^O). Section 5 illustrates that this approach is applicable to systems beyond d^0^ metals with two examples based on ^195^Pt and ^109^Ag NMR, starting again with molecular complexes before showing how chemical shifts can be used to elucidate the structure of immobilized molecularly defined catalysts, evaluate the environment of single-atom catalysts, and probe the surface acidity of oxide materials. In Section 6, we show how these methodologies can be extended to address complex issues with industrially relevant heterogeneous catalysts. On the example of Ziegler–Natta-type heterogeneous catalysts, which are complex hybrid organic–inorganic materials with low metal loading and are expected to exhibit a distribution of surface sites, we show in particular how ^51^V and ^47/49^Ti NMR analyses can complement each other and enable molecular-level understanding of the coordination environments of related V or Ti sites dispersed on MgCl_2_ surfaces, revealing details of the interaction of the transition-metal sites with the support. We conclude with the consideration of another important class of heterogeneous industrial catalysts, namely Ti-silicalite zeotypes, using ^47/49^Ti NMR.

Sections 4–6, devoted to specific case studies, first discuss the context and then illustrate the challenges and opportunities of this NMR-based approach before providing key highlights enabled by understanding NMR parameters of metal sites.

## 2 Experimental Measurements for Metal Nuclei and Basic Principles behind NMR Signatures

Modern solid-state NMR methods provide a suite of techniques for the measurement of CST and EFG parameters. Most essential are so-called magic-angle-spinning (MAS) measurement conditions, in which the sample is pneumatically rotated at the “magic angle” of 54.74° with respect to the direction of the magnetic field, in order to partially average inhomogeneous contributions to the NMR line shape, including CSA, internuclear dipole–dipole couplings, and the second-order component of the quadrupolar interaction. At slow MAS rates, the static powder pattern is broken up into a manifold of “spinning sidebands” separated by the frequency of rotation, ω_r_, with intensity focusing into the isotropic signal as the MAS rate is increased (Figure 1a). Thus, for spin 1/2 nuclei, CST parameters (δ_iso_, Ω, and κ) can be extracted in favorable cases by simulation of NMR lineshapes acquired at different MAS rates.

**Figure 1:**
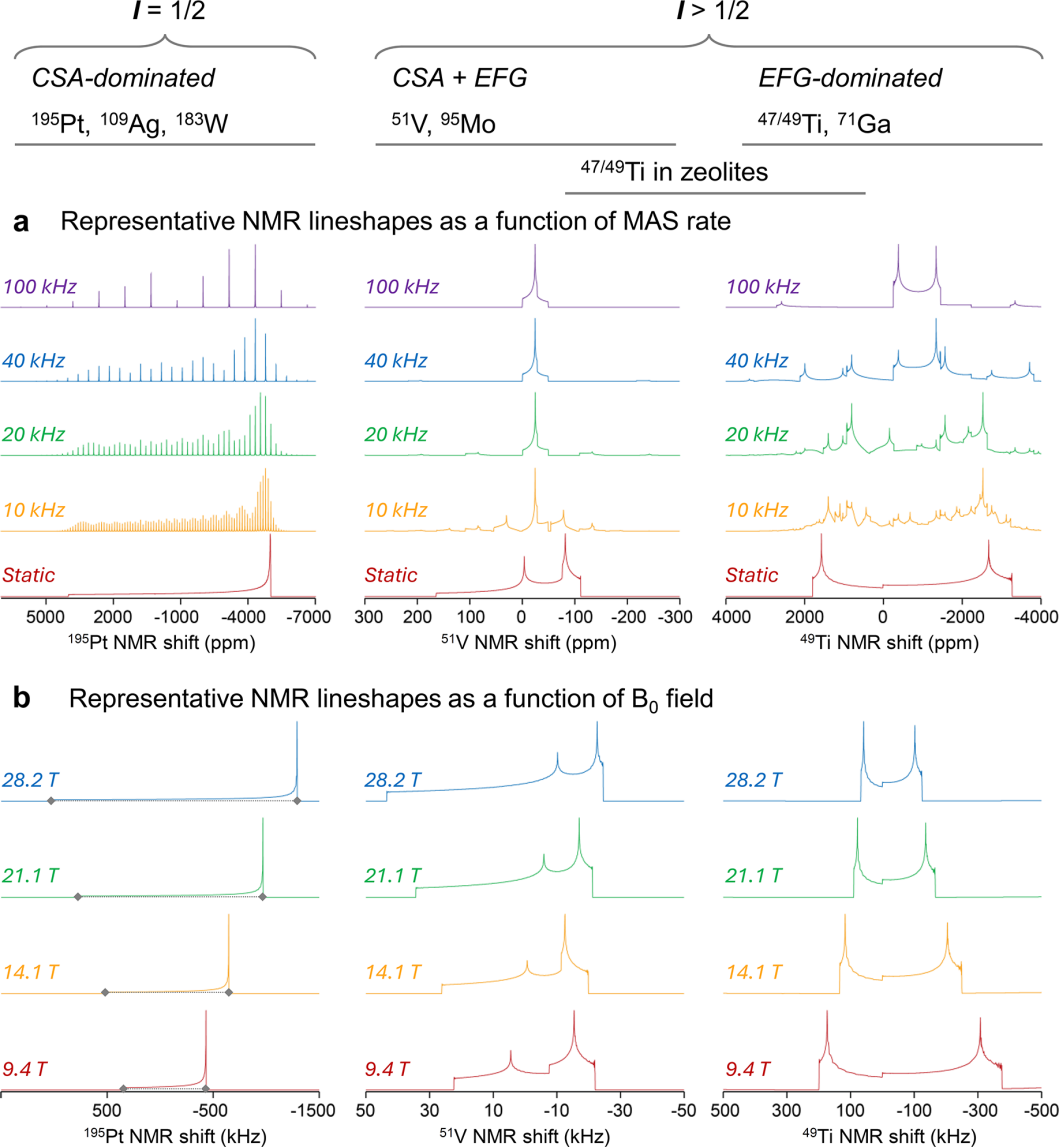
Simulated NMR lineshapes typical for spectra dominated by CSA (e.g., ^195^Pt), exhibiting the mixed effects of CSA and EFG (e.g., ^51^V), and dominated by EFG effects (e.g., ^49^Ti) as a function of (a) MAS rates and (b) B_0_ magnetic field strengths.

In heterogeneous solids, distributions of isotropic chemical shifts can overlap with spinning sidebands, complicating such line shape analysis. However, 2D solid-state NMR techniques such as magic-angle-turning (MAT)^39,40^ or phase adjusted spinning sideband (PASS),^41,42^ often used in combination with phase-incremented echo-train acquisition (PIETA),^43^ can be used to extract CSTs for individual sites.^44,45^

For quadrupolar nuclei (*I* > 1/2), line shape analysis is even more complicated as the second-order quadrupolar interaction is not fully averaged by MAS, yielding inhomogeneously broadened quadrupolar lineshapes even at very fast MAS rates (Figure 1a). This broadening can also be eliminated by mechanical rotation via double-orientation (DOR) or dynamic-angle-spinning (DAS) techniques,^46,47^ but these methods require (nowadays) uncommon and specialized instrumentation. An alternative is to separate isotropic and anisotropic contributions to quadrupolar lineshapes using 2D NMR methods, most commonly multiple-quantum MAS (MQMAS) techniques,^48–50^ which can resolve signals from different sites with distinct quadrupolar parameters and enable determination of their respective *C*_Q_ and η_Q_ values. As the magnitude of the second-order quadrupolar interaction is inversely proportional to magnetic field strength, the NMR spectra of half-integer quadrupolar nuclei exhibit a pronounced field dependence, and their quadrupolar parameters can be estimated by simulation of spectra acquired at different magnetic field strengths.

While the solid-state NMR techniques discussed above are now widely available and enable practical access to NMR parameters for more favorable nuclei, measuring the NMR signatures of transition-metal nuclei is often much more challenging. This is due to a number of complicating factors often present in combination for a given transition metal nucleus: very wide NMR chemical shift ranges, which can span up to tens of thousands of ppm; significant signal broadening related to large CSA and/or EFG effects; and low intrinsic sensitivities and/or natural isotopic abundances. These challenges are intrinsic to a given nucleus; NMR analyses of heterogeneous catalysts are often further complicated by distributions of chemical shifts related to surface inhomogeneity, as well as low metal loadings in industrially relevant catalysts.

Measuring and extracting CST and EFG parameters of transition-metal nuclei thus often require exploiting state-of-the-art methods to improve resolution and sensitivity. Given the dependence of the NMR signatures on the external magnetic field B_0_ (Figure 1b), it is appropriate to use low fields (<10 T) for species with large span (>1000 ppm) and negligible *C*_Q_,^51^ or high and ultrahigh fields (>20 T) for species with large *C*_Q_ values (>10 MHz).^50,52–57^ Note that higher magnetic field strengths provide intrinsic benefits to NMR signal sensitivity (due to the increase in the Boltzmann distribution of spin polarization) and resolution (due to chemical shift dispersion) provided access to MAS rates and radiofrequency pulse powers sufficient to effectively average CSA and excite the full chemical shift window of relevance. To overcome the threshold of low natural isotopic abundances, isotope labeling has been widely employed. Other methods to improve sensitivity and resolution include fast-MAS and indirect detection,^58–63^ cryogenic MAS conditions,^64,65^ dynamic nuclear polarization (DNP),^66–72^ and wide-line excitation and detection.^73–77^ These will be described in more detail in reference to the relevant case studies discussed below.

Another important measurement parameter is the sample temperature. Temperature-dependent dynamics of the metal center and associated ligands influence the CSA and EFG parameters through dynamic averaging. Measurements conducted at room temperature can yield lower observed *C*_Q_ values due to averaging as a result of thermal motion, making it difficult to compare experimental and computed values (the latter formally obtained at 0 K).^78^ One should note that the sample temperature inside the NMR rotor may deviate from temperatures measured in the stator near the rotor. The most reliable measurements of sample temperature use a “NMR thermometer”: NMR properties of a temperature-sensitive and receptive nucleus in an inert material like KBr^79^ or Pb­(NO_3_)_3_ ^80^ added to the NMR rotor. Finally, a large majority of transition-metal nuclei exhibit low gyromagnetic ratios, which intrinsically limits signal sensitivity, especially for the so-called “low-gamma” nuclei, typically classified as having resonance frequencies below ^15^N. Furthermore, the low corresponding Larmor frequencies are below the tuning ranges of most typical solid-state probes. Therefore, accessing these nuclei requires specially designed probes (Figure 2 and Table 1). As a consequence of these factors, experimental solid-state NMR studies of transition-metal nuclei have been limited, with fewer spectral data available for comparison to experimental results, particularly in the solid state.^81^

**Table 1:** Metal Nuclei Considered in This Perspective in Comparison with the Most Common NMR Nuclei (^1^H and ^13^C)

nucleus	nuclear spin *I*	natural abundance (NA), %	Larmor frequency at 14.1 T, MHz	electric quadrupole moment, fm^2^	gyromagnetic ratio γ, 10^7^·rad·T^–1^·s^–1^
^1^H	1/2	99.99	600.130		26.7511
^13^C	1/2	1.07	150.903		6.7283
^47^Ti	5/2	7.44	33.833	30.2	–1.5105
^49^Ti	7/2	5.41	33.842	24.7	–1.5109
^51^V	7/2	99.75	157.852	–4.3	7.0453
^95^Mo	5/2	15.90	39.110	–2.2	–1.7514
^109^Ag	1/2	48.16	27.927		1.2519
^183^W	1/2	14.31	25.004		1.1283
^195^Pt	1/2	33.83	129.009		5.8383

**Figure 2:**
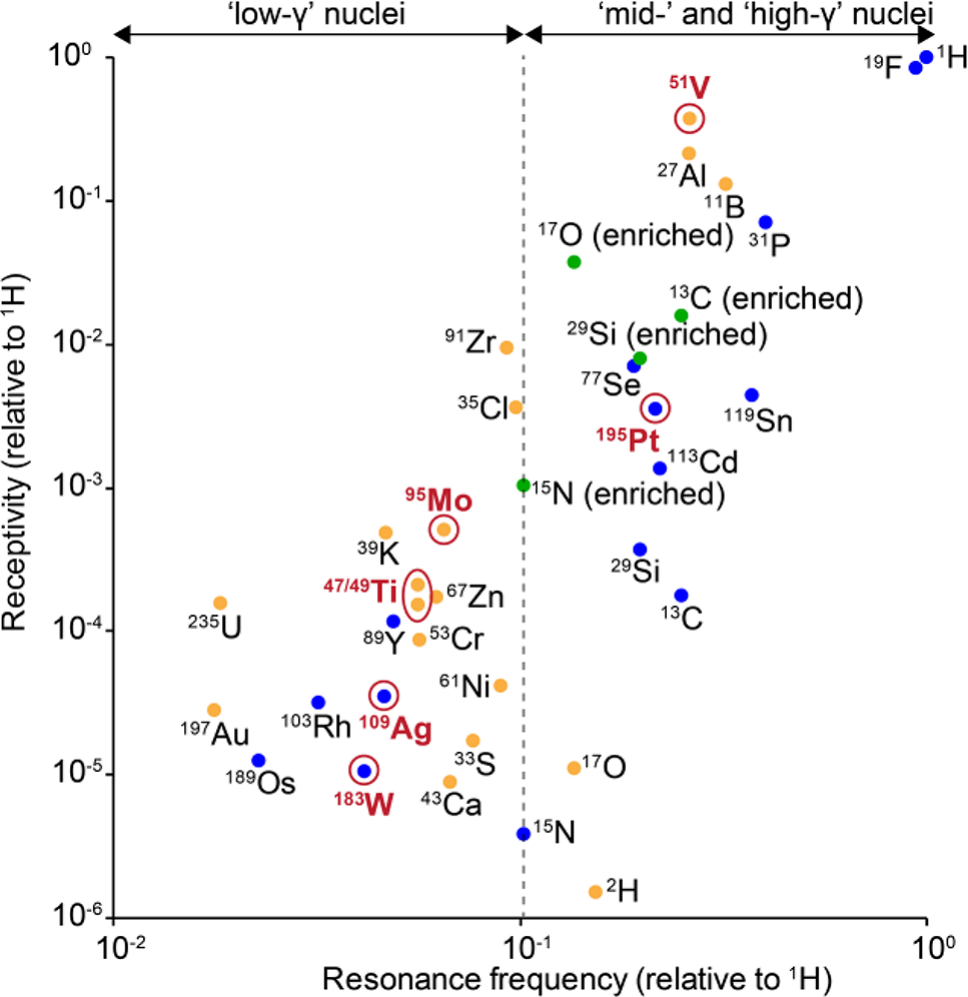
Log–log plot of relative NMR receptivity against relative resonance frequency for select NMR-active nuclei. Blue: *I* = 1/2 at natural abundance; yellow: *I* > 1/2 at natural abundance; green: receptivity at 100% abundance for nuclei that are commonly isotopically labeled. Nuclei discussed in this Perspective are colored red and highlighted with a circle.

## 3 Theoretical Background

Fundamental interpretation of experimentally measured chemical shifts and EFG tensors is greatly augmented by computational modeling and analysis. While chemical shifts can be experimentally determined with respect to a reference, chemical shielding values (σ) are obtained through calculations; they correspond to intrinsic properties of the nucleus and are referenced to the corresponding bare nucleus (e.g., C^6+^ for ^13^C). These computed shielding values require appropriate benchmarking, ideally on a series of compounds and materials using a linear regression approach across the relevant shift/shielding range.

The shielding tensors can be analyzed using the Ramsey formalism,^82^ where shielding is decomposed into diamagnetic (σ_*dia*_) and paramagnetic (σ_*para*_) contributions:

$$\sigma =\sigma_{\mathrm{dia}}+\sigma_{\mathrm{para}}$$

Diamagnetic contributions describe an isotropic screening and shielding of the nucleus from the external magnetic field due to the motion of core electrons and can be described as follows:

$$\sigma_{\mathrm{dia},\mathrm{ij}}=\frac{1}{2c^{2}}\sum_{k}\left\langle \Psi_{0}\right|\sum_{k}\frac{\left(r_{k}^{2}\delta_{\mathrm{ij}}-r_{k,i}r_{k,j}\right)}{r_{k}^{3}}\left|\Psi_{0}\right\rangle$$

where *i* and *j* denote the tensor elements (or directions of the tensor – *x*, *y*, *z*), Ψ_0_ denotes the ground-state wave function, *r*_*k*_ is the distance between the nucleus and the electron orbital *k*, *c* is the speed of light, and δ_*ij*_ is the Kronecker delta function (1 if *i* = *j*, 0 otherwise).

As the diamagnetic term arises mostly from core electrons, it is almost independent of the ligands bound to the observed nuclei. In contrast, the paramagnetic contribution that often governs the changes in CST leading to deshielding originates from couplings between occupied and vacant frontier molecular orbitals (FMOs) of adequate symmetry in the presence of the magnetic field as follows:

σpara,ij=−12c2∑n∑k⟨Ψ0|Lk,i^|Ψn⟩⟨Ψn|Lk,j^rk−3|Ψ0⟩En−E0+c.c.

where *E*_0_ is the energy of the energetic ground state, *E*_*n*_ and Ψ_*n*_ denote the energy and wave function of the *n* ^*th*^ excited state, respectively, and *c.c.* denotes the complex conjugate. When relativistic terms are considered, the paramagnetic contribution also includes spin–orbit coupling (SOC, σ_*SO*_). SOC is most pronounced for heavy atoms and is often treated in computation using a zeroth-order regular approximation (ZORA).^83–87^

As follows from the definition of the paramagnetic contribution, the deshielding of the nucleus (higher chemical shift values) increases with a decreasing energy gap between the two coupled FMOs of adequate symmetry. The states corresponding to these FMOs are “mixed” upon action of the angular momentum operator *L*_*k*_. Since the symmetry of *L*_*k*_ is the same as that of a rotational operator (*R*_*i*_), coupled orbitals are typically orthogonal to each other and the applied magnetic field, allowing for the use of a simple orbital rotation model and enabling the reconstruction of the electronic structure in an intuitive way, as shown in Figure 3. This principle establishes the foundation for natural chemical shift (NCS) analysis, which has been successfully applied to analyzing the origins of the chemical shift for a broad range of nuclei. Note that currently, although periodic calculations more accurately address structures of bulk materials, they are severely limited for analysis of the origins of chemical shieldings due to the difficulties associated with accounting for SOC, especially important for NMR calculations of heavy metal nuclei. In addition, chemical shift analysis relating to band structure is not yet routinely available.^88^

**Figure 3:**
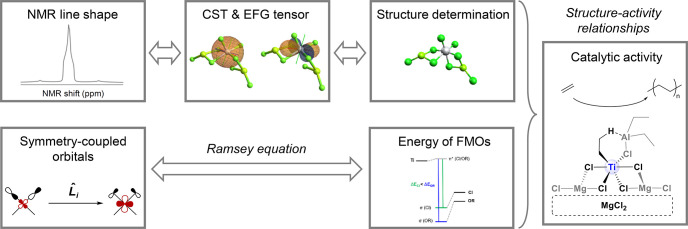
Concepts: from NMR signatures and lineshapes to NMR parameters, electronic structures, coordination environments, and structure–activity relationships.

Calculations of the EFG tensor involve finding its diagonal components (*V*_*11*_, *V*_*22*_, *V*_*33*_) at the site of the nucleus in its principal axis system and thereby calculating *C*_*Q*_ and η_*Q*_ parameters.^24^ These parameters are readily calculated for both cluster and periodic models and are implemented in most first-principles electronic structure calculations software packages. Computed *C*_*Q*_ values are often larger than those experimentally observed, due in part to the sensitivity of *C*_*Q*_ to dynamic averaging in experimental measurements, which are absent in calculations conducted at 0 K.^78^

The precision of the calculated chemical shift and *C*_*Q*_ values for most common nuclei is usually within the range of 5–10% of the ppm scale and 1–3 MHz, respectively, established on benchmarking sets of well-defined molecular compounds. Therefore, when comparing these parameters with experimental observations, it is important to always evaluate the confidence interval to avoid misassignment.

## 4 Early Transition Metals in d^0^ Configurations: From Molecularly Defined Systems to Supported Oxide Catalysts

Group 6 transition-metal elements in a d^0^ configuration (empty d-shell) such as Mo­(VI) and W­(VI) have broad applications in catalysis, in particular for the metathesis reactions of olefins and alkynes, two important reactions in organic synthesis.^89–94^ This section addresses the advantages and complementarity of the NMR parameters of the metal nuclei (^95^Mo) compared to those of associated ligands (^13^C) using a high-oxidation-state (d^0^) metal alkylidyne for alkyne metathesis catalysts as an illustrative example. Next, it shows that this approach can also be used to provide information about the key role of ligands in the corresponding W-based olefin metathesis catalysts. Finally, we look at silica-supported Mo-oxo-based olefin metathesis precatalysts, which lack organic ligands as facile spectroscopic probes, to show how the transition metal NMR parameters can provide direct descriptors for local geometry and associated reactivity of supported species.

### 4.1 Probing Frontier Molecular Orbitals and Understanding Alkyne Metathesis Activity from ^95^Mo vs ^13^C NMR

#### 4.1.1 Context

Alkyne metathesis has become a key reaction in organic synthesis that has attracted interest because of its atom economy and the progress in the design of highly efficient molecular catalysts.^91,95,96^ Recent studies of well-defined molecular Mo-based alkyne metathesis catalysts with the general formula (RO)_3_Mo≡CR’ (Figure 4a) have shown that the ^13^C chemical shift of the alkylidyne C atom increases with the fluorination of the alkoxide ligand (tBu_Fx_O, *x* = 0, 3, 6, and 9), driven mostly by the σ-donor strength of the monoanionic ligands. Orbital analyses of the ^13^C CSTs show that the chemical shift is driven by the energy of the vacant π*­(Mo≡C) orbitals, which are involved in the [2 + 2] cycloaddition between the alkylidyne and the alkyne, a key step of the catalytic cycle. Thus, the trends in the ^13^C chemical shift parallel the increase of alkyne metathesis activity for RO = ^*t*^BuF_0_O, ^*t*^BuF_3_O, and ^*t*^BuF_6_O. With RO = ^*t*^BuF_9_O, the catalyst becomes almost inactive due to the formation of a very stable metallacyclobutadiene intermediate, hence changing the rate-determining step and breaking the correlation between activity and chemical shift (Figure 4b).^97^ Of particular interest are the class of highly active and functional-group-tolerant “canopy catalysts” bearing tripodal silanolate ligands.^98^ However, no correlation of the ^13^C chemical shift with catalytic reactivity could be found for silanolate-supported catalysts. Furthermore, in contrast to the alkoxide series, there is no clear trend between the isotropic ^95^Mo and ^13^C chemical shifts of such catalysts, measured in solution (Figure 4b). The complex having RO = Ph_3_SiO, for instance, exhibits an alkylidyne carbon ^13^C chemical shift similar to that of the *^t^* BuF_3_O complex, but with markedly different ^95^Mo chemical shifts (Figure 4b). The different trends in ^13^C and ^95^Mo NMR characteristics thus suggested that the (fluoro)­alkoxide and silanolate complexes belong to different catalyst classes. Additionally, they suggested that ^95^Mo chemical shifts encode additional information and that a more comprehensive understanding of electronic structure and reactivity could be obtained from analysis of NMR parameters of both ligand and metal nuclei.

**Figure 4:**
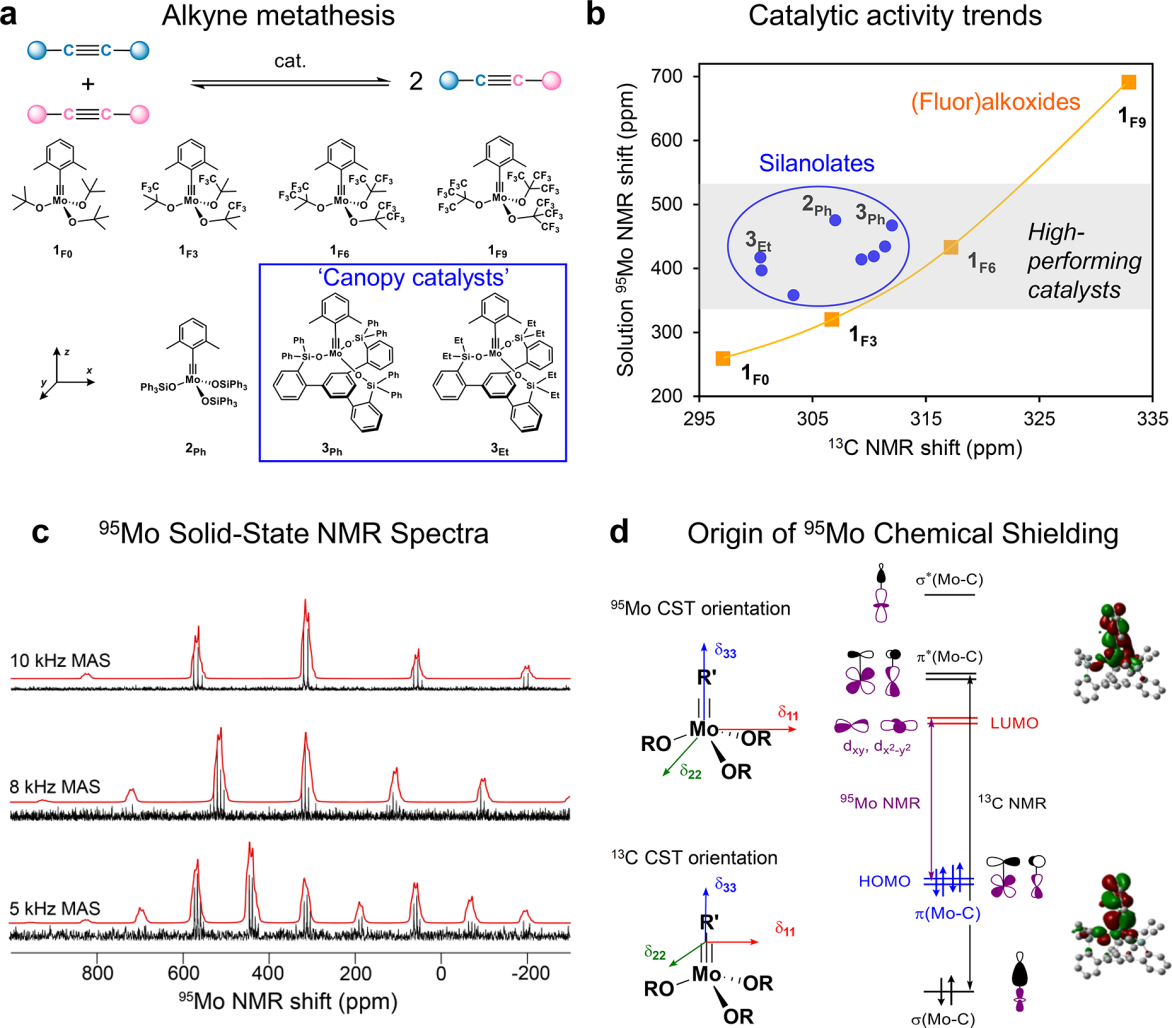
Solid-state ^95^Mo NMR analysis of molecular Mo alkylidyne alkyne metathesis catalysts: (a) alkyne metathesis reaction and representative Mo alkylidynes with (fluoro)*tert*-butoxy or silanolate ligands, including the high-performing podate canopy catalysts, (b) trends in ^95^Mo and ^13^C δ_*iso*_ in solution, (c) representative solid-state ^95^Mo NMR spectra of a tris­(tertbutoxy) Mo alkylidyne at different MAS rates (14.1 T, 100 K) and corresponding line shape analysis, and (d) CST orientations and most important orbital couplings contributing to ^95^Mo and ^13^C chemical shifts/shieldings.

#### 4.1.2 NMR Challenges and Opportunities

Mo has two NMR-active isotopes, ^95^Mo and ^97^Mo, which are both low-gamma and quadrupolar. ^95^Mo has more favorable NMR properties for measurement because of its higher natural abundance (15.92%), similar Larmor frequency (−1.7514 × 10^7^ rad s^–1^ T^–1^), and smaller quadrupolar moment (−2.2 fm^2^) compared to ^97^Mo (9.56%, −1.7884 × 10^7^ rad s^–1^ T^–1^, and 25.5 fm^2^, respectively). To increase ^95^Mo NMR signal sensitivity and mitigate dynamic averaging of chemical shift and EFG parameters, ^95^Mo NMR solid-state spectra can be acquired at low temperature (ca. 100 K) and moderate magnetic field strengths (14.1 T) by using a quadrupolar Carr–Purcell–Meiboom–Gill (QCPMG) detection scheme.

#### 4.1.3 Highlights

Analysis of the ^95^Mo solid-state NMR spectra of a series of Mo alkylidynes, whose lineshapes are dominated by CSA due to the rather small *C*_Q_ (2–5 MHz), enabled extraction of CST parameters and estimation of *C*_Q_ values (Figure 4c) for a series of well-defined Mo alkylidyne compounds having either fluoroalkoxide or silanolate X ligands, including representative high-performing podate canopy catalysts. The ^95^Mo δ_iso_ values increase with fluorination of the alkoxide ligands, correlating to ligand σ-donor strength as observed in solution. Furthermore, the ^95^Mo δ_iso_ is determined mostly by the principal components that lie perpendicular to the Mo≡C bond, denoted as δ_⊥_ = (δ_11_ + δ_22_)/2. Due to the symmetry of these compounds, the ^95^Mo CST thus manifests both the strength of the Mo≡C bond and the influence of the orthogonal Mo–O bonds. Indeed, orbital analyses of the ^95^Mo CSTs show that δ_⊥_ arises primarily from the coupling of high-lying π­(Mo≡C) orbitals with low-lying σ*­(Mo–O) orbitals, which correspond to HOMO and LUMO, respectively, of the Mo alkylidynes. This sharply contrasts with the alkylidyne ^13^C CSTs, which have a similar orientation but are driven by the coupling of σ­(Mo≡C) orbitals and low-lying π*­(Mo≡C) orbitals. These results establish that the ^95^Mo NMR parameters manifest more directly the frontier molecular orbitals (HOMO/LUMO) involved in reactivity compared to those obtained from ^13^C NMR. The ^95^Mo CST analyses furthermore illustrate the unique influences of the podate silanolate ligands: though similar to the ^*t*^Bu_F6_O ligand in terms of σ-donor strength, the architectures of the different silanolate ligands enforce specific ligand geometries and corresponding Mo-ligand π-interactions. In the case of the podate canopy catalysts, this enables partial delocalization of the HOMO and LUMO across the aromatic ligand substituents, which raises the energy of the HOMO, leads to particularly deshielded ^95^Mo NMR signatures, and provides a better overall electronic match for [2 + 2] cycloaddition reactions with alkynes, the first step in the catalytic cycle of alkyne metathesis (Figure 4d). Overall, ^95^Mo NMR parameters provide a direct link to reactivity and, in this case, information on the relative positions of the HOMO and LUMO and the HOMO–LUMO gap that explains trends in catalyst activity and offer descriptors for targeted catalyst design.^27^

### 4.2 Understanding the Role of Ligands in the Stability of Reaction Intermediates in Olefin Metathesis from ^183^W NMR Parameters

#### 4.2.1 Context

Olefin metathesis, like alkyne metathesis, involves [2 + 2] cycloaddition as a key elementary step. This step requires first coordination of the substrate olefin to the metal alkylidene sites to generate the putative olefin adduct intermediate (Figure 5a). In Schrock-type metathesis catalysts of general formula (X)­(Y)­M­(E)­(=CHR) with M = Mo or W in d^0^ configuration and E = oxo or imido, while proposed to be a key step, rate determining, and highly influenced by the σ-donating X,Y-ligands, the associated olefin complex (with a trigonal bipyramidal (TBP) geometry, the olefin *trans* to X and the Y ligand sharing the basal plane with the alkylidene and E) remains elusive. Recent efforts have enabled the isolation of a surrogate cationic species, [W­(=CH_2_)­(=O)­Cl­(IMes)_2_]­[OTf], (M = W, E = oxo, X = IMes, Y = Cl), where the olefin reactant is substituted by an additional isolobal NHC fragment, as olefins and IMes are formally both σ-donor and π-acceptor ligands and can be viewed as isolobal entities. Using ^183^W NMR, it was possible to understand the role of the Y-ligand in modulating the electronic structure and, thereby, the reactivity of this key reaction intermediate.

**Figure 5:**
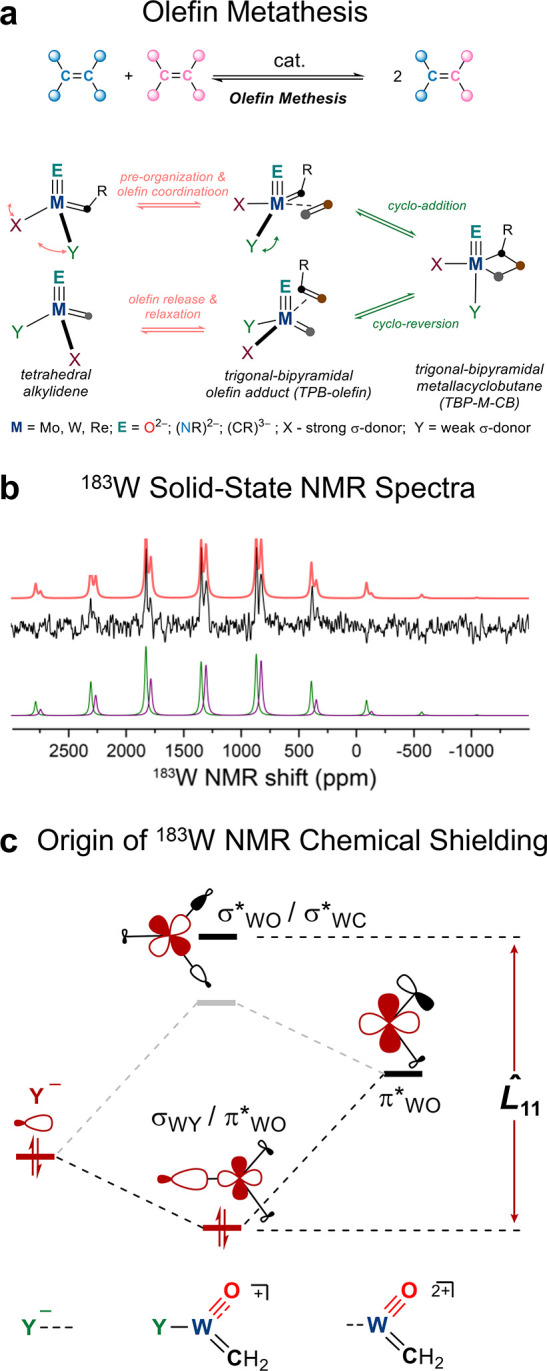
(a) Olefin metathesis and key elementary steps, (b) ^183^W­{^1^H} solid-state CPMAS NMR (9.4 T, 94 K, 5 kHz MAS) of the [W­(=CH_2_)­(=O)­Cl­(IMes)_2_]­[OTf] molecular complex, and (c) most important orbital couplings contributing to ^183^W chemical shieldings.

#### 4.2.2 NMR Challenges and Opportunities

Tungsten possesses a single NMR-active spin 1/2 nucleus, ^183^W with low NA (14.31%), which displays an extremely low γ (γ = −1.12070 × 10^7^ T^–1^ s^–1^), long nuclear spin–lattice relaxation times, a broad chemical shift range (exceeding 10,000 ppm), and large anisotropy. All of these factors complicate the acquisition of ^183^W NMR spectra, especially in the solid state. While solution ^183^W NMR spectra have already shown that the isotropic chemical shift provides potent information about the local structure including the formal oxidation state,^99^ the corresponding solid-state NMR studies have been exceedingly rare, mostly limited to proofs-of-concept and method development focused on well-defined bulk model systems.^100–102^ However, state-of-the-art solid-state ^183^W NMR methods, in particular indirect ^1^H detection and cryogenic MAS conditions, have recently been shown to provide high ^183^W NMR signal sensitivity for well-defined W compounds, enabling facile analysis of molecular species in the solid state.^60,103^

#### 4.2.3 Highlights

[W­(=CH_2_)­(=O)­Cl­(IMes)_2_]­[OTf], an olefin complex surrogate, displays a noteworthy solid-state ^183^W NMR signature (Figure 5b): a very large CSA with one very deshielded principal component (δ_11_), already indicating the presence of low-lying vacant orbitals in this complex with a TBP geometry. NCS analysis reveals that the deshielding of δ_11_ is driven by the nature of the three basal ligands, σ­(W=C), σ­(W=O), and σ­(W–Cl), that compete for the same two d-orbitals (d_x2‑y2_ and d_*xy*_).

Notably, similar ^183^W NMR signatures are calculated for the corresponding putative olefin intermediate, [W(=CH_2_)(=O)Cl(IMes)(C_2_H_2_)]^+^, confirming the isolobal nature of IMes and olefin. Extending the computational study by substituting Y = Cl for Me, O^*t*^Bu, and O^*t*^Bu_F9_ further highlights the importance of this basal ligand in modulating the electronic structure: the ^183^W NMR signatures show that the ancillary σ-donating ligand (Y) influences the hybridization of the competing O and CH_2_ ligands. Namely, moving from weak to strong σ-donor Y-ligand (O^*t*^Bu_F9_ < O^*t*^Bu < Cl < Me) decreases the triple bond character of the oxo ligand (from sp to sp^2^). Such a decrease in the M=O bond order results in a higher energy barrier for the structural reorganization from tetrahedral to TBP geometry. This increases the energetic cost of olefin coordination, corroborating earlier theoretical studies,^104^ and reveals the molecular-level origins of this ligand effect (Figure 5c). In fact, this change in electronic structure and hybridization of the oxo-ligand can also be detected in the computed ^17^O NMR signatures. Strong σ-donor monoanionic ligands, such as methyl, lower the skew value of ^17^O CST, indicating significant M=O double bond character, while weaker σ donor ligands retain high ^17^O skew values, reflecting the triply bonded nature of the oxo-ligand. Overall, the ^183^W NMR allows probing and experimentally benchmarking metal-centered orbitals, which is complementary to the ligand centered technique, giving access to the interplay of effects of different ligands on the metal center.

### 4.3 Establishing Structure–Activity Relationships in Mo-Oxo-Based Silica-Supported Olefin Metathesis Catalysts

#### 4.3.1 Context

Olefin metathesis is an industrial process used, for instance, for the on-purpose production of propylene. Industrial catalysts are based on silica-, alumina-, or aluminosilica-supported Mo or W oxides, but it is widely accepted that these catalysts generate *in situ* Mo or W alkylidenes that are structurally analogous to the well-defined molecular Schrock alkylidenes discussed above (Figure 6a).^89,105^ However, the active species have never been observed and are likely present at very low quantities under typical reaction conditions.^106^ Thus, there is still much interest in understanding the formation of the active species (activation pathways) and what specific characteristics of metal oxo sites drive this process.^106–108^ For instance, significant differences in catalytic activities have been observed depending on metal loading and preparation methods across a series of silica-supported Mo oxides, suggesting that particular configurations of surface Mo are more prone to being activated.^109^ Silica-supported Mo oxo sites with similar Mo loadings prepared by impregnation versus surface organometallic chemistry (SOMC) show markedly different reactivity patterns after activation under reducing conditions for both gas- and liquid-phase metathesis.^27,108,110^ This is also evident in the titration of reducible Mo sites with the two-electron organosilicon reductant 1-methyl-3,6-bis­(trimethylsilyl)-1,4-cyclohexadiene (denoted as **red-1**, Figure 6b). Reducible Mo sites are ca. 6 times more prevalent in an SOMC-derived catalyst compared with catalysts prepared via impregnation.

**Figure 6:**
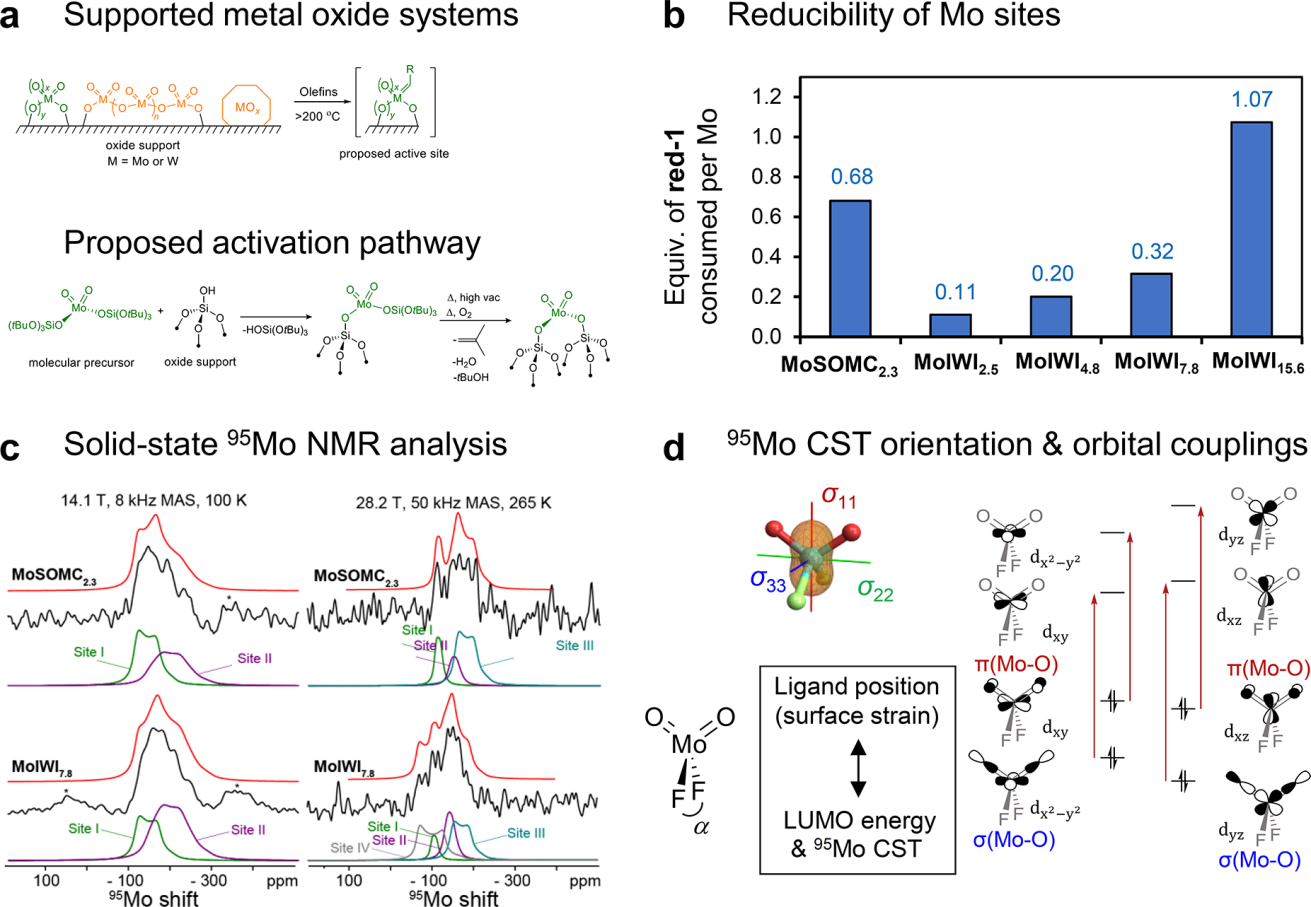
(a) Supported Mo oxides as olefin metathesis (pre)­catalysts and proposed activation pathway. (b) Fraction of reducible Mo sites for SOMC-derived (**MoSOMC**_**2.3**_) and impregnation-derived (**MoIWI**_*x*_) silica-supported Mo oxo (pre)­catalysts with different Mo loadings determined by titration with a molecular organosilicon reductant. (c) Solid-state ^95^Mo NMR spectra of **MoSOMC**_**2.3**_ and **MoIWI**_**7.8**_ measured at 14.1 T, 100 K, and 8 kHz MAS or 28.2 K, 265 K, and 50 kHz MAS, with ^95^Mo line shape deconvolutions. (d) ^95^Mo CST orientation and relevant orbital couplings for a model Mo dioxo complex.

Despite these marked reactivity differences, SOMC- and impregnation-derived supported Mo oxo precatalysts exhibit nearly identical UV–vis, FTIR, and Mo K-edge X-ray absorption near edge (XANES) spectra, all consistent with the presence of isolated Mo­(VI) dioxo surface sites. As the Mo oxo precatalysts lack accessible organic ligands to act as spectroscopic handles, and given the utility of ^95^Mo NMR analyses for deciphering reactivity trends discussed above, solid-state ^95^Mo NMR has been used to identify the electronic structure and distinguish Mo sites in order to draw structure–activity relationships.

#### 4.3.2 NMR Challenges and Opportunities

In addition to the challenges of solid-state ^95^Mo NMR discussed in reference to the Mo alkylidynes above, measurements of supported Mo oxo species are additionally complicated. Mo sites supported on amorphous silica often have low weight loadings of Mo (typically 1–3 wt % for submonolayer surface coverage, depending on the surface area of the support) and exhibit intrinsic inhomogeneous distributions of chemical shifts arising from distributions of surface sites (heterogeneity). Furthermore, the low symmetry of surface species can lead to large *C*_Q_ or CSA and correspondingly broad line widths (though CSAs for Mo oxo species are typically relatively small). The advent of ultrahigh magnetic field spectrometers,^111^ and especially commercial 28.2 T NMR magnets with fast-spinning MAS probes,^112^ improves ^95^Mo sensitivity and resolution sufficiently to enable analysis of dilute Mo sites in supported Mo oxo catalysts and extraction of their relevant NMR parameters.^113^

#### 4.3.3 Highlights

Notably, in contrast to Mo K-edge XANES, very different ^95^Mo NMR signatures are observed for catalysts generated by SOMC or impregnation approaches. Analyses of solid-state ^95^Mo NMR spectra acquired at 14.1 and 28.2 T establish the presence of at least four distinct types of ^95^Mo species with different δ_iso_ and *C*_Q_ values (Figure 6c): Site I (δ_iso_: −95 ppm, *C*_Q_: 3.3 MHz); Site II (−127 ppm, 4.0 MHz); Site III (−135 ppm, 6.1 MHz); Site IV (−47 ppm, 7.3 MHz). Sites III–IV required 28.2 T measurement conditions to be observed, highlighting the necessity for high-field NMR. Notably, the relative intensity of the particularly deshielded and low-*C*_Q_^95^Mo NMR signal from Site I correlates with both high metathesis activity and high reducibility of the isolated Mo­(VI) sites. Indeed, for a series of molecular Mo mono-oxo compounds, the ^95^Mo chemical shift was found to correlate directly to reducibility of the metal site due to its relation to ligand σ-donor strength.^114^ In the case of the silica-supported Mo oxo (pre)­catalysts, however, all spectroscopic evidence points to the presence of Mo dioxo species in predominately tetrahedral geometries, suggesting that small geometric perturbations account for the remarkably different ^95^Mo NMR signals of Sites I–IV (*vide infra*).

As reduction of Mo­(VI) to Mo­(IV) is proposed as a first step in the generation of catalytically active Mo alkylidenes from Mo oxo sites, the link between ^95^Mo NMR parameters and reducibility could provide a potent probe for the reactivity of specific surface sites. The response of the ^95^Mo δ_*iso*_ and *C*_Q_ to ligand geometry and coordination number was screened using first-principles calculations of small model analogues for Mo surface sites of the general formula [(F_3_SiO)_2_Mo­(O)_2_(L)_*x*_], where L is a neutral coordinating ligand and *x* is 0–2. Based on the computational analysis, Site I was attributed to a Mo­(VI) dioxo disiloxide site with a highly strained tetrahedral geometry, generated in a higher proportion by SOMC-based syntheses than by impregnation. For such structures, NCS analysis showed that the ^95^Mo δ_iso_ values are driven mostly by coupling of occupied σ­(Mo–O) and π­(Mo–O) NLMOs to low-lying virtual orbitals composed of Mo d-orbitals, including LUMO. These virtual orbitals respond strongly to the position of the monoanionic ligands due to an overlap of ligand p-orbitals and Mo d-orbitals. Decreasing the SiO-Mo­(O)_2_–OSi angle (increasing strain) leads to a deshielded ^95^Mo chemical shift due to the reduced energies of the LUMO (and other low-lying virtual orbitals), which also leads to and explains the increased reducibility of strained sites (Figure 6d). By comparison, Raman spectroscopy and Mo XANES are mostly insensitive to the configurations of Mo dioxo sites because the local structure of the [Mo­(O)_2_] fragment is almost invariant as a function of the positions of the monoanionic ligands. These subtle differences in local structure explain why solid-state NMR can resolve differences that are indistinguishable by other techniques. The ^95^Mo NMR parameters thus provide direct, unique, and site-specific insights into the structure–activity relationships of different types of surface sites, key descriptors for improved catalyst design.^27^

## 5 NMR Signatures of Late-Transition and Coinage Metal Elements: ^195^Pt and ^109^Ag

This section examines two examples based on late-transition metals with a partially or completely filled d-shell. First, ^195^Pt NMR is used to understand electronic structures from molecular compounds to a novel class of single-atom catalysts. Second, NMR is used to establish an acidity scale based on the ^109^Ag NMR chemical shift, enabling determination of the Bro̷nsted acidity and speciation of surface sites in materials.

### 5.1 ^195^Pt: Challenges and Opportunities of a Large CSA in Understanding Surface Sites

#### 5.1.1 Context

Pt is ubiquitous in catalysis: from molecular Pt complexes used in homogeneous catalytic processes to the corresponding immobilized species. Furthermore, beyond the realm of supported Pt nanoparticles, the corresponding single-atom catalysts (SACs) have recently appeared as a novel class of materials with notable reaction properties across (thermo-, photo-, and electro-)­catalysis and ideal metal usage.^115^ Yet, for these “heterogeneous” systems and in contrast to their homogeneous counterparts, understanding the Pt coordination environment with atomic precision (Figure 7a) remains challenging due to the low concentration of Pt sites (down to 1 Pt wt % and below) and the presence of a distribution of surface species. Considering that this information is not accessible via classical characterization techniques such as X-ray absorption spectroscopy (XAS) and X-ray photoelectron spectroscopy (XPS), solid-state ^195^Pt NMR spectroscopy (Figure 7b,c) is a promising approach to understanding the coordination environments of Pt centers across a range of Pt-based catalysts, from immobilized Pt complexes to SACs (Figure 7d).

**Figure 7:**
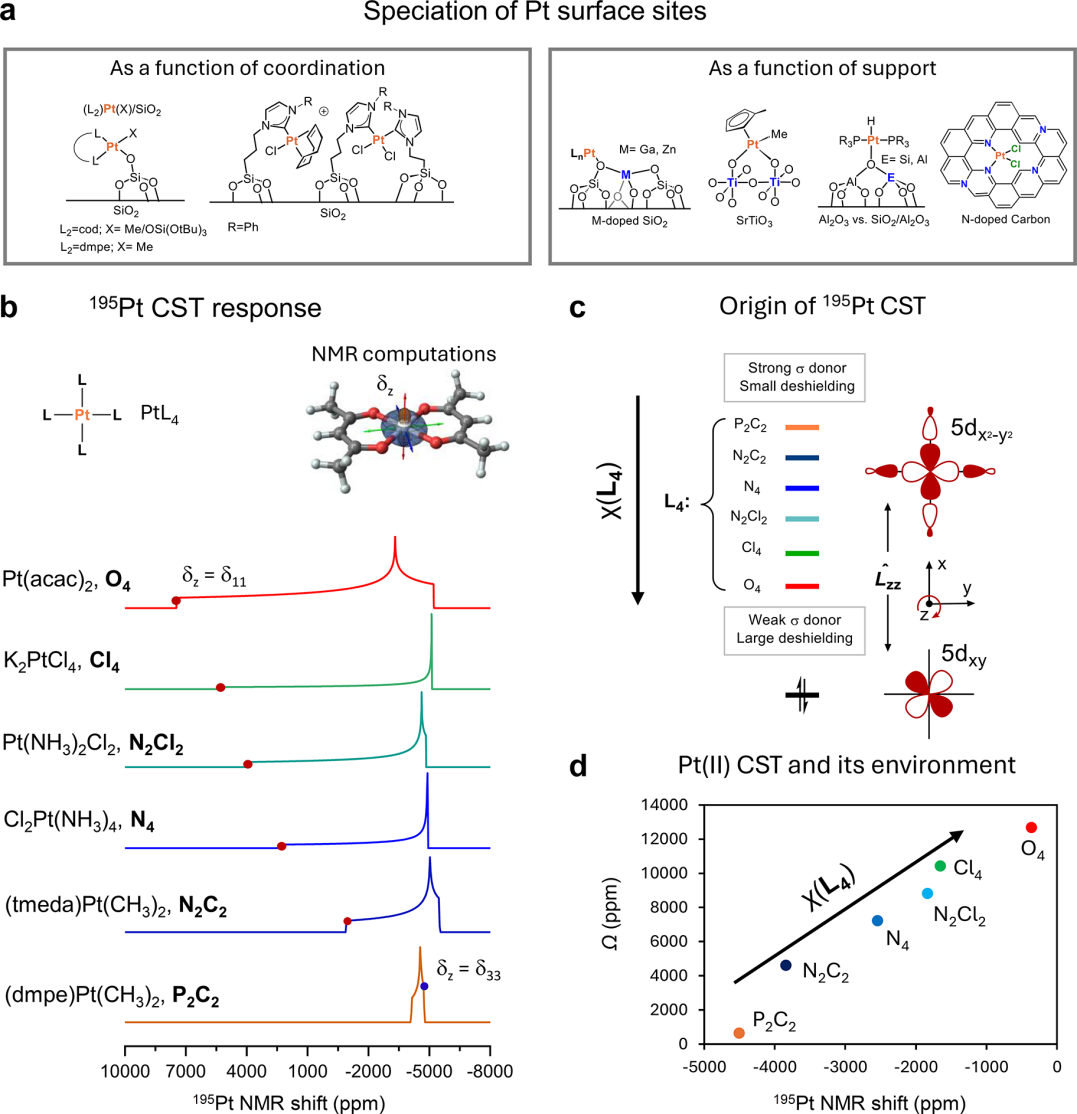
(a) Characterization of supported Pt species as a function of their ligand environment and support. (b) Computed CST for a series of Pt­(II)­L_4_ complexes with varying ligand sets. δ_*z*_ is the principal component of the CST that is aligned with the *z*-axis of the molecular frame. The position of δ_*z*_ is highlighted on each ^195^Pt powder pattern to illustrate its dependence on the ligand environment. A 3D Pt CST for Pt­(acac)_2_ is shown (δ_11_: red, δ_22_: green, δ_33_: blue). (c) Orbital origin of ^195^Pt CSA in Pt­(II)­L_4_ complexes. The orbital (Pt_*xy*_–L/Pt_*x*2‑*y*2_–L) responsible for the deshielding along the Z-component of the CST is shown, as well as the influence of the ligand environment of the vacant Pt_*x*2‑*y*2_–L orbital. (d) Typical δ_*iso*_/Ω values for the Pt­(II) complex as a function of their ligand environment.

#### 5.1.2 NMR Challenges and Opportunities

Pt has one NMR-active isotope, ^195^Pt (33.8%, γ = 5.768 × 10^7^ T^–1^ s^–1^). Although its NMR signal receptivity is about 20 times that of ^13^C (at natural abundance), the acquisition of ^195^Pt solid-state NMR is considerably more challenging because of very large possible CSAs, reaching >13,500 ppm for some systems.^116–118^ Acquisition of the full ^195^Pt solid-state NMR line shape is difficult, but, if possible, it can provide valuable information on the (electronic) structure of the metal center because it is highly sensitive to the formal oxidation state and coordination environment, as shown in pioneering studies involving in particular some of the first use of chemical shift analysis to trace back the origin of differences in CSTs (*vide infra* for more information).^29,119^ Regarding sensitivity enhancement, several approaches under both static and MAS conditions have been developed for solid-state ^195^Pt NMR, involving both direct detection methods such as DNP-enhanced CP-CPMG,^120,121^ broadband adiabatic inversion cross-polarization (BRAIN-CP),^122^ wideband uniform-rate smooth truncation (WURST)-CPMG,^123,124^ and indirect ^195^Pt detection through NMR-active “spy-nuclei” (^1^H, ^13^C, ^31^P) with resonance-echo saturation-pulse double-resonance (RESPDOR) or heteronuclear multiquantum correlation (HMQC) pulse sequences.^118,125–127^ Additionally, progressive saturation of the proton reservoir (PROSPR) has been shown to be efficient on cisplatin and thus could be of potential interest for analyzing surface species.^128,129^ These methodologies enabled the acquisition of NMR signatures of Pt single-sites in MOFs, organometallic Pt sites supported on SiO_2_-based supports, Al_2_O_3_, and SrTiO_3_, and more recently SACs on carbon-based supports.^74,118,121,130–134^ Notably, DNP enhancements could be successfully achieved in combination with these NMR approaches; this allowed for even higher sensitivity and faster acquisition of the ^195^Pt NMR lineshapes.^121,125,131,133,134^

#### 5.1.3 Highlights

The ^195^Pt δ_iso_ is very sensitive to the ligand environment of Pt in any common oxidation number (0, II, IV).^118,121,127^ Additionally, the ^195^Pt CSA is also responsive to the geometry around the metal center (square planar vs octahedral), with octahedral Pt­(IV)­L_6_ species featuring relatively isotropic ^195^Pt NMR signatures (Ω < 2000 ppm), with δ_iso_ varying with the nature of the ligands.^135^

Pt­(II)­L_4_ species display very specific NMR signatures related to their square-planar geometry, leading to axially symmetric ^195^Pt NMR CSTs with Ω values reaching 13,500 ppm.^118^ NMR calculations show that the ^195^Pt δ_iso_ and Ω values are mostly driven by the principal component perpendicular to the plane made by the ligands, the most deshielded (δ_*z*_ = δ_11_), while δ_//_ hardly varies (Figure 7b).^29,119^ NCS analysis shows that the deshielding of δ_*z*_ arises from the magnetic coupling of Pt 5d orbitals, specifically between the occupied Pt_*xy*_–L (π-Pt), typically considered nonbonding, and the empty (σ*-Pt) orbitals associated with Pt_*x*2‑*y*2_–L. Considering that the energy of the σ* is mostly driven by the nature of L, the more electronegative the element bound to Pt, the lower the σ* and the greater the deshielding of δ_*z*_. Hence, δ_iso_ and Ω values increase for PtL_4_ complexes in the order L = *P* < C < *N* < Cl < O-based ligands (Figure 7d).^29,119^ Note that deshielding can also be modulated by π-effects, which affect the energy of the nonbonding Pt_*xz*_ and Pt_*yz*_ orbitals: π-donor ligands raise the energy of the filled nonbonding orbitals and decrease the gap with the σ* orbital, leading to increased deshielding and more positive chemical shifts (π-acceptor ligands have the exact opposite effect leading to shielding).^118^

With this knowledge in hand, solid-state ^195^Pt NMR becomes an invaluable tool to understand the coordination environment of Pt in immobilized catalysts. Regarding SiO_2_-supported Pt­(II) square-planar species prepared through grafting, the ^195^Pt CSA in materials containing down to 0.015 wt % Pt was accessed via indirect acquisition from spy nuclei (^1^H, ^13^C, ^31^P).^118,125^ A correlation between the observed changes in the ^195^Pt NMR signatures and the metal coordination environment can be drawn via the aforementioned orbital rotational model, allowing determination of the coordination environment of the Pt center down to an atomic level (Figure 7a).^118^ Similarly, ^195^Pt NMR CSA parameters complemented by ^13^C δ_*iso*_ values and *J*_Pt–C_ coupling constants enabled determination of the coordination environment of immobilized Pt­(II) species prepared by grafting on the NHC ligands of hybrid organosilica materials. In particular, it was possible to determine the presence of two distinct surface species, arising from different immobilization mechanisms of the metal precursor onto the support.^133^ This methodology has also been extended to other supports (Figure 7b).^135^ ^195^Pt NMR was applied on SrTiO_3_-supported Pt sites to confirm their oxidation state and coordination environment. More recently, the formation of different Pt–H sites upon oxidative addition of a Pt(0) diphosphine complex to OH groups in SiO_2_ and SiO_2_/Al_2_O_3_ was proven by analyzing their Pt fingerprints.^134^

Most recently, the use of ultrawide line solid-state ^195^Pt NMR has been shown to enable recording the NMR signatures of ^195^Pt sites in SACs.^138^ The fitting of the experimental lineshapes through Monte Carlo simulations resulted in a distribution of Ω and δ_*iso*_ parameters (with expectation values of <Ω> and <δ_*iso*_>), consistent with the presence of several coordination environments consisting of C, N, Cl atoms, and combinations thereof. The visualization of the fitting parameters in Ω/δ_*iso*_ correlation plots (Figure 7d) allowed to assess the homogeneity of the Pt chemical environments in a sample, showing the most abundant coordination environment. Additionally, the direct comparison of the ^195^Pt signatures of two samples made it possible to evaluate the reproducibility of synthetic protocols as well as compare materials with different Pt loadings and even supports. Ultimately, this approach was used to follow the evolution of the Pt coordination environment under catalytic conditions, monitoring the catalyst design process from ^195^Pt solid-state NMR.

The current solid-state ^195^Pt NMR methods allow for the acquisition of full CSTs of the metal centers in single-site and single-atom catalysts as well as the determination of their (electronic) structures and distributions thereof. This knowledge can be applied for the preparation of (heterogeneous) catalysts with specific electronics and reactivity.

### 5.2 ^109^Ag: Acidity Scale and Probing Surface Bro̷nsted Acid Sites on Materials

#### 5.2.1 Context

For catalyst materials, evaluating the strength and speciation of surface hydroxyl groups (−OH) and their Bro̷nsted acidity is critical but remains a very challenging task. Due to the lack of spectroscopic descriptors and distributions of (surface) −OH sites, there is no convenient method for the direct determination of the Bro̷nsted acidity of specific −OH sites in materials. Considering that protons are formally isolobal to coinage metals in their +1 oxidation state, Cu­(I), Ag­(I) and Au­(I) with a s^0^d^10^ configuration, one can foresee that the chemical shift of isolobal Ag­(I) complexes with various anionic X ligands could provide direct information on the p*K*_a_ of that protonate ligand (X–H) (Figure 8a).

**Figure 8:**
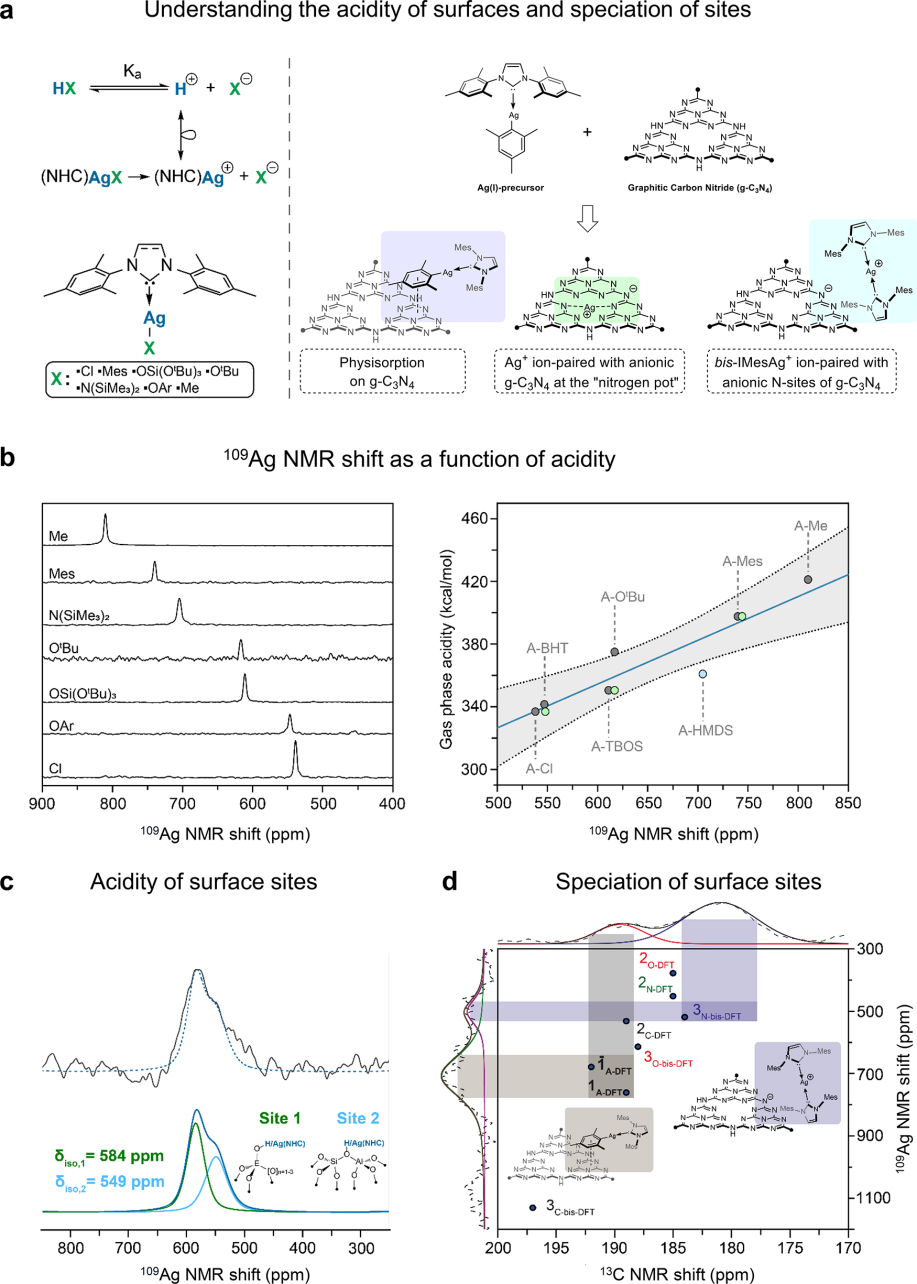
(a) (Left) Isolobal analogy between Bro̷nsted acid H-X and the corresponding (NHC)­Ag-X complex and the series of (NHC)­Ag-X complexes used for the ^109^Ag NMR study. (Right) Proposed grafting mechanism and resulting surface species generated upon grafting of (NHC)Ag-Mes with g-C_3_N_4_. (b) (Left) The solution ^109^Ag NMR spectra of **SIMesAg-X** complexes (**A** series). (Right) Linear correlations between δ^109^Ag and calculated gas-phase acidity. Gray circles indicate **A** series, and green circles represent unsaturated **IMesAg-X** complexes (**B** series).^136^ (c) (Top) Experimental spectrum of (NHC)­Ag@Si/γ-Al_2_O_3_. (Bottom) Fitted spectrum showing isotropic chemical shifts of 584 and 549 ppm (decomposition shown in green and light blue). The sites associated with the signals are shown next to the respective signals (E = Si/Al).^136^ (d) 2D correlation of computed ^13^C and ^109^Ag chemical shifts among different possible surface sites in (NHC)­Ag@g-C_3_N_4_. The dotted and solid lines represent the experimental and simulated spectra of (NHC)­Ag@g-C_3_N_4_, respectively. The region of intersection predicts the surface sites as shown beside.^137^

#### 5.2.2 NMR Challenges and Opportunities

Ag has two isotopes with a 1/2 nuclear spin, namely, ^107^Ag (γ = −1.0828 × 10^7^ T^–1^ s^–1^, NA 51.8%) and ^109^Ag (γ = −1.2449 × 10^7^ T^–1^ s^–1^, NA 48.2%). The preferred isotope for NMR spectroscopic observation is ^109^Ag, with a slightly higher receptivity. However, the observation of ^109^Ag NMR signals has always been a challenge due to the rather low γ and extremely long spin–lattice relaxation times. Hence, relatively few ^109^Ag NMR studies have appeared in the literature.^139–141^ With the recent popularization of commercial probes for observing low-gamma nuclei and development of DNP surface-enhanced NMR spectroscopy (DNP-SENS),^66,67,142,143^ ^109^Ag NMR has been increasingly applied to probe the detailed local structure of various materials in order to correlate their functionalities and the structural properties.^136,144–149^

#### 5.2.3 Highlights

Notably, the ^109^Ag NMR chemical shift across a series of (NHC)­Ag-X complexes has been shown to linearly correlate with the p*K*_a_ (or gas phase acidity) of the corresponding Bro̷nsted acid (H–X) (Figure 8b). This correlation originates from the fact that σ-donation of the X-type ligand is related to the electronegativity of X and thereby the calculated gas-phase acidity and p*K*a values of the conjugate acid (H–X). Considering that the ^109^Ag NMR chemical shift can be used as a quantitative descriptor for Bro̷nsted acidity of X–H groups, this strategy has been further explored to measure the p*K*_a_ of surface OH groups across oxide supports by grafting (NHC)­Ag–Mes and measuring the solid-state ^109^Ag NMR spectra of the corresponding grafted species. Each type of supported Ag species can provide a specific and distinct NMR response, thereby giving both the speciation and the p*K*_a_ of surface sites (Figure 8c). Based on this strategy, it has been found that Si/γ-Al_2_O_3_ exhibits some strong Bro̷nsted acid sites, associated with a δ_iso_(^109^Ag) of 549 ppm, which markedly differs from what is observed for SiO_2_ and γ-Al_2_O_3_ with δ_iso_(^109^Ag) of 569 and 598 ppm, respectively. The increased acidity of that site can be linked to the presence of pseudobridging silanol sites, where a silanol interacts with a neighboring unsaturated Lewis acidic Al site. These results are consistent with the low-temperature ^15^N CP-MAS NMR of samples exposed to ^15^N-pyridine as a separate probe of Bro̷nsted acidity.

Furthermore, this approach can be used to elucidate the nature of surface sites in nonoxide materials such as graphitic carbon nitride (*g*-C_3_N_4_) using (NHC)­Ag-Mes as a reactive spectroscopic probe (Figure 8a). While microscopy analyses clearly show atomically dispersed Ag, ^109^Ag NMR in combination with ^13^C NMR and DFT calculations show that the primary surface sites include *bis*-NHC-Ag^+^ coordinated with N-sites, strongly adsorbed (NHC)Ag molecular precursor interacting with *g*-C_3_N_4_, and Ag^+^, likely situated in the “nitrogen pot”, providing new insights into the surface chemistry of functionalized *g*-C_3_N_4_ materials (Figure 8d).^137^

## 6 NMR Signatures of Metal Sites in Industrially Relevant Catalysts

### 6.1 Learning about Subtle Geometrical Changes in Surface Sites and Support Effects in Ziegler-Natta Catalysts through NMR Lineshapes

#### 6.1.1 Context

Understanding the structure and properties of surface metal sites in industrially relevant catalysts represents further challenges due to the complex nature and the heterogeneity of surface sites in heterogeneous catalysts. Notable examples are Ziegler–Natta (ZN)-type catalysts comprised of metal sites, typically Ti but also other metals like V, interacting with a MgCl_2_ support and promoted by additional organic ligands.^150,151^ While studied for more than 60 years, the development of next-generation ZN catalysts remains essentially empirical as little is known about the nature of the surface sites at various stage of the catalyst preparation (Figure 9a, pre- and postactivated catalysts).^152–154^ Recent work involving NMR and EPR spectroscopy of the activated catalysts has shown that Ti­(III) bimetallic alkyl species are generated upon activation, confirming that pretreatment chlorination with BCl_3_ helps to generate these sites.^155^ However, the structures of the surface Ti sites remain unknown prior to the activation step, making metal NMR analyses a method of choice.

**Figure 9:**
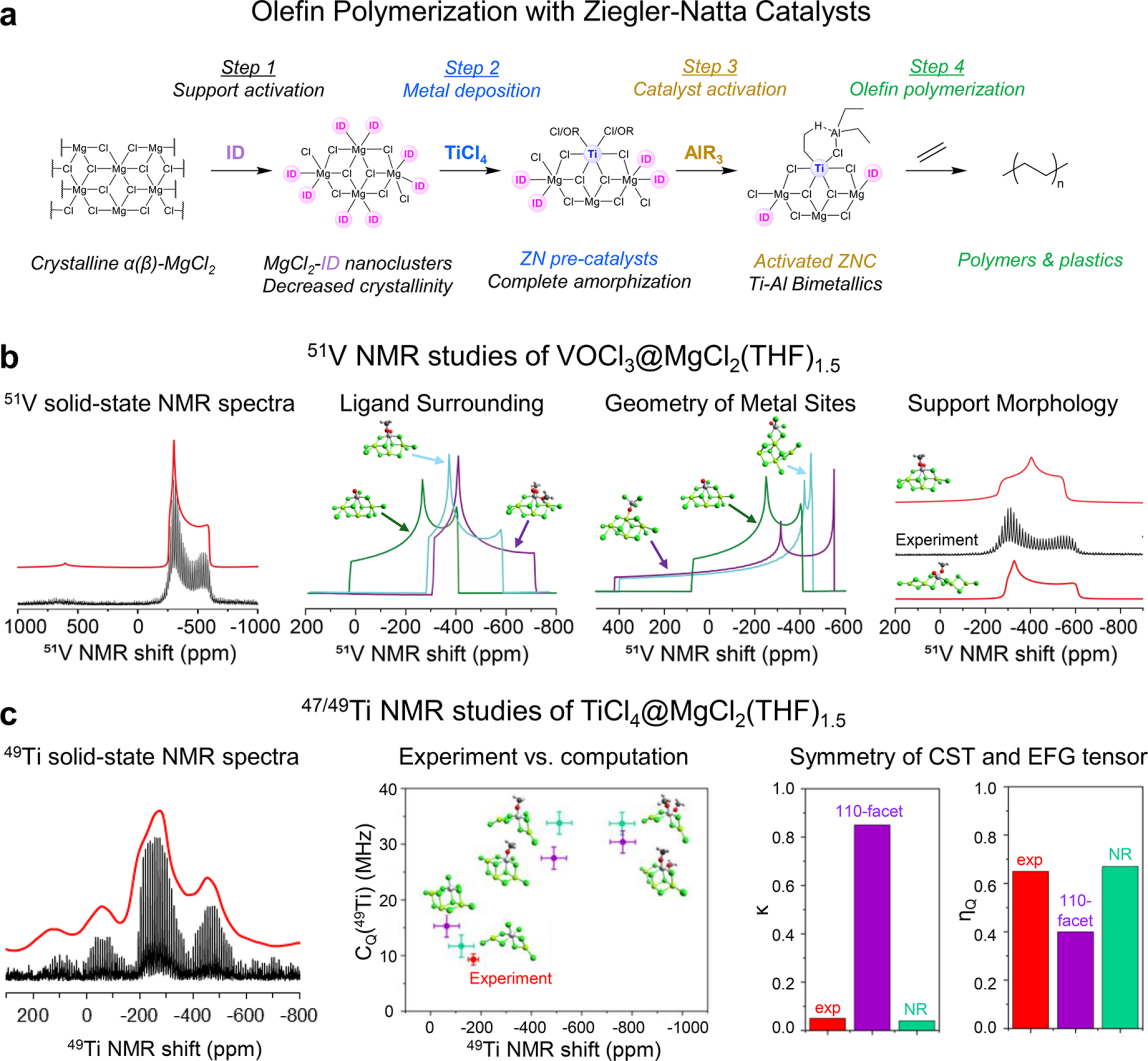
Understanding the preactive sites in ZN catalysts: (a) typical steps of preparation of ZN catalysts, (b) ^51^V solid-state NMR spectrum of the V-based ZN catalyst (VOCl_3_@MgCl_2_(THF)_1.5_) and its interpretation on the molecular level using DFT computations, and (c) ^47/49^Ti solid-state NMR spectrum of the ZN precatalyst (TiCl_4_@MgCl_2_(THF)_1.5_) and its interpretation on the molecular level.

#### 6.1.2 NMR Challenges and Opportunities

Solid-state ^47/49^Ti NMR spectroscopy has thus far been very limited because of the low intrinsic NMR sensitivity of Ti isotopes, which is due to a combination of factors (low-γ nuclei, small receptivity, large quadrupolar coupling constant), coupled with the low industrially relevant weight loadings of Ti in heterogeneous catalysts (typically ca. 0.5–2 wt %). Additionally, Ti possesses two NMR-active nuclei, ^47^Ti (*I* = 5/2, NA 7.44%) and ^49^Ti (*I* = 7/2, NA 5.41%), whose gyromagnetic ratios are so similar that NMR signals from ^47^Ti and ^49^Ti usually overlap (Table 1). Notably, Ti can be substituted by V in ZN-catalysts, and the latter displays much more favorable NMR properties: ^51^V (NA > 99%) posseses nuclear spin with multiplicity (*I* = 7/2), but with a rather low quadrupolar moment (−5.2 fm^2^) and high gyromagnetic ratio (−7.0455139 × 10^7^ rad s^–1^ T^–1^), making ^51^V NMR rather accessible (receptivity 0.384 relative to ^1^H, which is 2 orders of magnitude higher than that of ^49^Ti). Here, we show how isostructural substitution of one nucleus (^47/49^Ti) by another more receptive one (^51^V) can help solve the structure of surface sites in complex materials such as ZN catalysts.

#### 6.1.3 Highlights

Using first V-based ZN catalysts based on VOCl_3_ dispersed on MgCl_2_, VOCl_3_/MgCl_2_(THF)_1.5_, solid-state ^51^V NMR spectra show a very well-defined signature, highlighting the presence of one specific type of vanadium site. In particular, the small quadrupolar moment enables extraction of the CST parameters. DFT modeling augmented by NCS analysis allows refining the structure, namely, coordination geometry, the specific ligands bound to V, and their specific positions, thereby providing information about the local morphology of the support. In short, the ^51^V NMR signature indicates that the observed V sites are hexacoordinated and contain V-oxo species with a terminal alkoxy ligand along with four μ^2^-bridging surface chloride ligands. Furthermore, the specific orientation and shape of the CST, almost axially symmetric with κ value close to 1, best describes a V site adsorbed on a disordered surface, modeled as a nanoribbon-like MgCl_2_ support,^156,157^ In contrast, V sites with the same ligand set but adsorbed on the 110-facet of a crystalline MgCl_2_ support, as was previously proposed in the literature,^158,159^ would yield a nonaxially symmetric CST (Figure 9b). Furthermore, NCS analysis helps to establish the origin of this NMR signature and CST (δ, Ω, and κ). In short, the span (Ω), driven mostly by δ_11_, originates from the number of terminal X-anionic ligands (coordination number) which alters the dominant orbital coupling form V–Cl σ-bonds *cis* to the V–O_oxo_ bond that couple with low lying π*­(V–Cl) for multiple terminal chlorides to the respective coupling between the π­(V–Cl_terminal_) to the respective σ*­(V–Cl) for the model bearing only one terminal ligand. Therefore, hexacoordinated V species with only one terminal X ligand display a lower δ_11_ and smaller Ω, close to the experimental findings. Furthermore, the isotropic chemical shift is highly dependent on the electronegativity of the ligands bound to V and points to the presence of one alkoxide ligand. Finally, κ, associated with the relative position of δ_22_ vs δ_11_ and δ_33_, depends on the relative position of the anionic ligands and is thus influenced by the support morphology originating from the difference in the energy gap between π­(V–O_oxo_) and σ*­(V–O_oxo_), which is smaller for the amorphous surface model.^157^

With this information in hand and advances in both hardware and pulse sequences, it was also possible to tackle the challenge of identifying Ti sites in ZN precatalysts. The use of high magnetic field strengths (21.1 T) in combination with low temperature measurement conditions (100 K) and CPMG detection, allowed obtaining ^47/49^Ti NMR signatures of a BCl_3_-treated Ti-based ZN catalyst that exhibited superior activity in ethylene polymerization due to BCl_3_ treatment.^160^

Although the Ti sites in BCl_3_ treated ZN precatalysts are intrinsically different to V sites in VOCl_3_/MgCl_2_ (V-oxo vs Ti-chlorido), some parallels could be found especially when focusing on the homogeneity and crystallinity of the MgCl_2_ support. Like the V analogues, the structure of observed Ti sites has indeed been found to also be well-defined and to correspond (based on DFT calculations) to a fully chlorinated hexacoordinated Ti species adsorbed on a distorted surface of the MgCl_2_ support (Figure 9c). The ^47/49^Ti isotropic chemical shifts allow distinction between the ancillary ligands and are found to be related to the electronegativity of the ligand, similar to ^51^V. The NMR signatures of the fully chlorinated Ti sites parallel their increased activity in ethylene polymerization, observed upon activation of the precatalyst, hinting that these sites are prone to activation by alkyl aluminum leading to the formation of the active species. Moreover, the experimentally obtained *C*_Q_, η_Q_, and κ parameters point toward the absence of an axial symmetry, which is in better agreement with Ti sites interacting with a distorted nanoribbon-like MgCl_2_ surface.^161^

Notably, both V- and Ti-based ZN precatalysts are best described as exhibiting well-defined surface structures, whereas the active ZN catalysts are known to be multisite polymerization catalysts. This information is of great particular importance as it helps establish that the variety and complexity of Ti sites are likely introduced in the activation step rather than intrinsic in the precatalyst itself.

### 6.2 Titanosilicalite-1: What Can We Learn from ^47/49^Ti NMR

#### 6.2.1 Context

Zeolites, i.e., crystalline nanoporous aluminosilicates, represent a highly important class of heterogeneous catalysts due to numerous industrial applications in multiton processes such as cracking, isomerization, alkylation, and aromatization.^162^ The framework composition of zeolites has been extended to so-called “zeotypes”.^163^ While zeotypes have structures similar to zeolites, tetrahedral sites can be occupied by a variety of elements other than Si and Al. Notably, titanosilicate (Ti-)­zeotypes are an important class of heterogeneous catalysts that have enabled the development of sustainable industrial oxidation processes (epoxidation, ammoximation, etc.) (Figure 10a).^164,165^ Since the discovery of titanosilicalite-1 (TS-1) as an oxidation catalyst ca. 40 years ago, the active site has been debated, a result of using predominantly empirical correlations across IR and UV resonance Raman spectroscopies^166–168^ based on ill-defined or nonsuitable references, e.g., heterogeneous compounds or molecular models without siloxide ligands.

**Figure 10:**
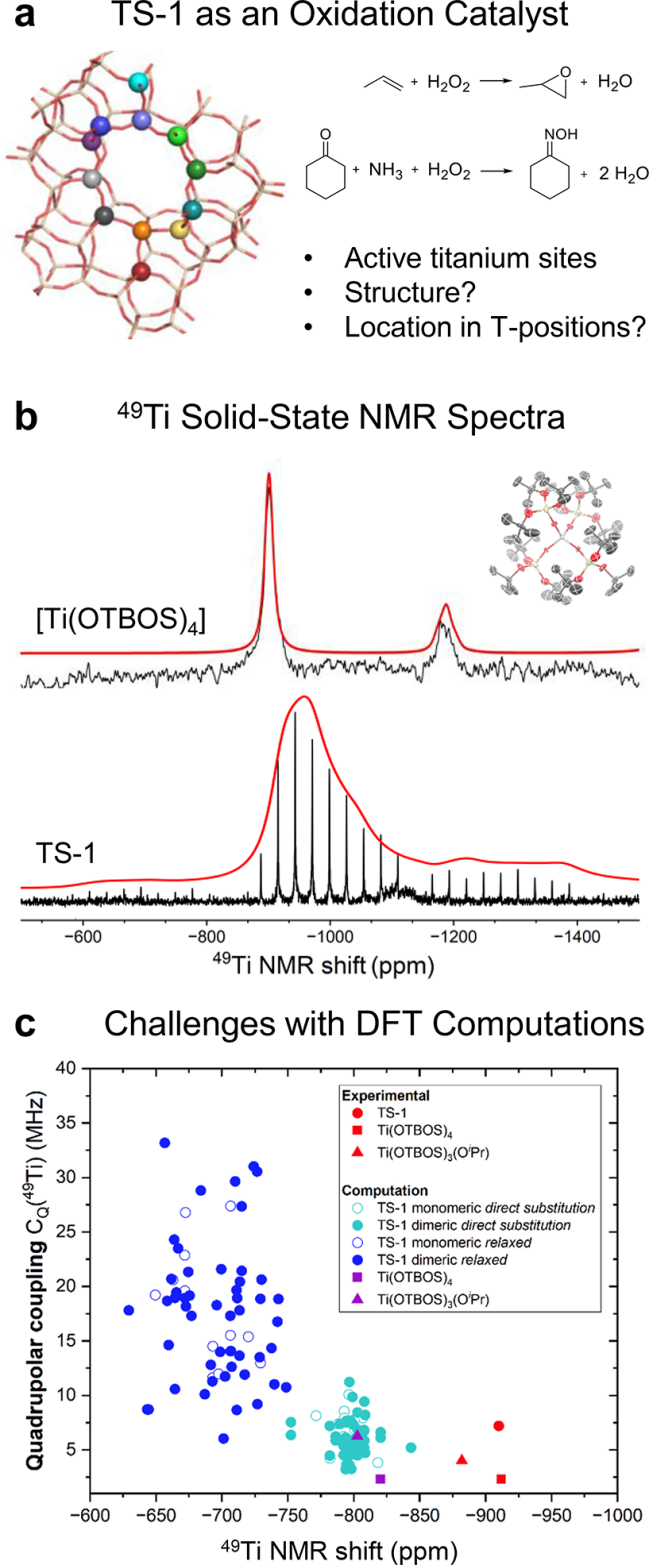
(a) Structure of TS-1 with highlighted distinct T-positions and open research questions. (b) Solid-state ^47/49^Ti NMR spectroscopic signatures of TS-1 and [Ti­(OTBOS)_4_] (as well as its measured crystal structure). (c) Computed ^47/49^Ti NMR parameters for a variety of T-site models.

#### 6.2.2 NMR Challenges and Opportunities

Preliminary theoretical simulations have shown that for typical Ti-zeotype materials, ^47/49^Ti signals with small CSA and low to intermediate C_Q_s (up to 10 MHz) can be expected. Solid-state ^47/49^Ti NMR measurements hence require either ultrahigh magnetic fields (>28.2 T) or high magnetic fields in combination with intermediate magic angle spinning rates^112^ to resolve the solid-state ^47^Ti and ^49^Ti NMR spectroscopic signatures. Thanks to the recent development of a low-γ NMR probe with cryogenic cooling of electronic components at high magnetic field (18.8 T) and MAS capabilities,^169^ it was indeed possible to record the solid-state ^47/49^Ti NMR spectroscopic signature of TS-1 despite the low Ti weight loading (1.5 wt %) of industrially relevant formulations.^64^

#### 6.2.3 Highlights

The measured solid-state ^47/49^Ti NMR spectroscopic signature of TS-1 shows a ^49^Ti NMR chemical shift of −910 ppm and a *C*_Q,max_(^49^Ti) of 7.2 MHz (as obtained from the Czjzek model consistent with a distribution of EFGs),^170^ consistent with an all-oxygen (slightly) distorted-tetrahedral first coordination sphere. Comparison with the molecular homologue [Ti­(OTBOS)_4_] (TBOS = tris­(*tert*-butoxy)­siloxy) with almost perfectly tetrahedral Ti sites indeed shows pronounced differences with respect to the quadrupolar coupling constant (*C*_Q_(^49^Ti) = 1.8 MHz, Figure 10b).

Notably, both TS-1 and [Ti­(OTBOS)_4_] show an almost identical Ti K-edge XANES pre-edge,^171^ highlighting the power of solid-state ^47/49^Ti NMR of resolving small differences in local symmetry. Detailed computational studies suggest that the measured quadrupolar coupling in TS-1 could be used to pinpoint which T-positions in TS-1 are occupied by Ti; however, at this stage, periodic DFT calculations are not accurate enough to reproduce the experimental data (Figure 10c). To limit the obtained uncertainty, it is required to include effects related to local vs periodic structural parameters – notably unit cell expansion upon Ti substitution and the effect of temperature-induced structural dynamics. While unit cell expansion could be benchmarked on lattice parameters obtained from X-ray diffraction,^172^ structural dynamics could be limited by measuring the solid-state ^47/49^Ti NMR spectra at low temperatures (>100 K).^173,174^ Analyzing the possible temperature-induced changes in the spectroscopic signature could also give further insight into the structural flexibility of a given Ti-zeotype.

## 7 Conclusions and Outlook

Overall, this Perspective highlights the unique information derived from NMR parameters of transition-metal nuclei, namely, chemical shift and EFG tensors, which can be directly linked using first-principles calculations to electronic structure, local geometry, and specific FMOs. Extracting these parameters requires a suite of solid-state NMR techniques, which are now easier to access because of ongoing advances in NMR instrumentation and methodologies that provide dramatic enhancements in solid-state NMR signal sensitivity and resolution. These enable measurement of a growing array of low-gamma nuclei that have previously been largely inaccessible. Included in this category are nuclei of many transition-metal elements with important roles in catalytic transformations, which can now be accessed for both well-defined molecular systems and heterogeneous catalysts at catalytically relevant loadings. Our analyses of the NMR signatures of such metal nuclei show that their NMR characteristics are complementary to spy nuclei (nuclei of ligand atoms bound to the metal sites); in fact, metal NMR signatures are especially sensitive because of their specific FMO energies (due to the smaller difference of energy between occupied and vacant metal-based MOs). Transition-metal NMR parameters in particular are driven by the HOMO and/or the LUMO, provided that they have suitable symmetry. Thus, NMR signatures of transition-metal nuclei provide information distinct and complementary to those of main group nuclei found in their ligands. In addition, because of the symmetry of the magnetic moment operator, NMR gives information about electronic structures complementary to other spectroscopic techniques and particularly suited to connect to reactivity and establish structure–activity relationships by connecting frontier orbitals of σ- and π-symmetry. Indeed, as transition metals are frequently the active sites in catalysis, their NMR parameters can provide valuable descriptors for understanding and predicting chemical reactivity. With the continuous development and progress of experimental NMR techniques, we expect growing opportunities to use NMR signatures and descriptors in predictive catalysis but also in solving longstanding problems in heterogeneous catalysis and material chemistry, where unravelling the structures of surface sites and reaction intermediates remains a contemporary challenge. An example is the suite of “NMR crystallography” approaches, which are also growing in scope and sophistication,^175–178^ where NMR parameters (often in combination with XRD and DFT computation) are leveraged for structure determination.

Catalyst formulations relevant to established and emerging industrial processes often exhibit different types and complex distributions of supported metal species (isolated sites, clusters, nanoparticles) that evolve over treatment/reaction conditions. For such materials, unraveling the origin of reactivity requires deploying multiple spectroscopic, microscopy, and scattering techniques when possible under operating conditions. Integrating transition-metal NMR parameters offers routes to directly determine 3D atomic structures and site-specific reactivity of surface species and defects relevant to catalysis and other applications. While focused on homogeneous and heterogeneous catalytic systems, transition metals and their coordination environments are central to numerous applications, from the closely related field of metalloenzymes, all the way to device applications (e.g., semiconductors, light emitting and adsorbing devices), where one could link NMR parameters to physical properties and functions. A key to the success of this approach relies on the development and improvement of NMR methodologies including computational approaches for periodic and challenging (e.g., paramagnetic) systems.

## References

[1] Hartwig, J. F. Organotransition metal chemistry: from bonding to catalysis; University Science Books: 2010.

[2] Blasco T. (2010). Insights into
reaction mechanisms in heterogeneous catalysis revealed by in situ
NMR spectroscopy. Chem. Soc. Rev..

[3] Haw, J. F. In-situ spectroscopy in heterogeneous catalysis; Wiley-VCH: 2002.

[4] Niemantsverdriet, J. W. Spectroscopy in catalysis: an introduction; Wiley-VCH: 2007.

[5] Van Doorslaer, S. ; Murphy, D. M. EPR Spectroscopy in Catalysis. In EPR Spectroscopy: Applications in Chemistry and Biology; Springer: 2012; pp 1–39. 10.1007/128_2011_237 21928011

[6] Lunsford, J. H. EPR Methods in Heterogeneous Catalysis. In Catalysis: Science and Technology; Springer: 1987; pp 227–256.

[7] Reven L. (1994). Solid-state
NMR studies of supported organometallics. J.
Mol. Catal..

[8] Hunger, M. Solid-State NMR Spectroscopy. In Zeolite Characterization and Catalysis: A Tutorial; Springer: 2009; pp 65–105.

[9] Grekov D., Vancompernolle T., Taoufik M., Delevoye L., Gauvin R. M. (2018). Solid-state
NMR of quadrupolar nuclei for investigations into supported organometallic
catalysts: scope and frontiers. Chem. Soc. Rev..

[10] Pregosin, P. S. NMR in organometallic chemistry; Wiley-VCH: 2012.

[11] MacKenzie, K. J. D. ; Smith, M. E. Multinuclear Solid-State Nuclear Magnetic Resonance of Inorganic Materials; Elsevier Science: 2002.

[12] Bell, A. T. NMR Techniques in Catalysis; Taylor & Francis: 1994.

[13] Spiess H.
W. (1997). Multidimensional
solid state NMR: A unique tool for the characterisation of complex
materials. Berichte der Bunsengesellschaft für
physikalische Chemie.

[14] Qi G., Wang Q., Xu J., Deng F. (2021). Solid-state NMR studies
of internuclear correlations for characterizing catalytic materials. Chem. Soc. Rev..

[15] Zheng A., Li S., Liu S.-B., Deng F. (2016). Acidic Properties and Structure–Activity
Correlations of Solid Acid Catalysts Revealed by Solid-State NMR Spectroscopy. Acc. Chem. Res..

[16] Li S., Lafon O., Wang W., Wang Q., Wang X., Li Y., Xu J., Deng F. (2020). Recent Advances of Solid-State NMR
Spectroscopy for Microporous Materials. Adv.
Mater..

[17] Reif B., Ashbrook S. E., Emsley L., Hong M. (2021). Solid-state NMR spectroscopy. Nat. Rev. Methods
Primers.

[18] Gioffrè D., Florian P., Pigeon T., Raybaud P., Chizallet C., Copéret C. (2025). Classification
and Identification of Facet and Edge
specific γ-Al2O3 Surface Sites from 1H/27Al NMR Cross-Signatures
and DFT modelling. J. Am. Chem. Soc..

[19] Polenova T., Gupta R., Goldbourt A. (2015). Magic Angle Spinning NMR Spectroscopy:
A Versatile Technique for Structural and Dynamic Analysis of Solid-Phase
Systems. Anal. Chem..

[20] Porat-Dahlerbruch G., Goldbourt A., Polenova T. (2021). Virus Structures and Dynamics by
Magic-Angle Spinning NMR. Annu. Rev. Virol..

[21] Abragam, A. The Principles of Nuclear Magnetism; Clarendon Press: 1961.

[22] Slichter, C. P. Principles of Magnetic Resonance; Springer: 1996.

[23] Herzfeld J., Berger A. E. (1980). Sideband intensities
in NMR spectra of samples spinning
at the magic angle. J. Chem. Phys..

[24] Autschbach J., Zheng S., Schurko R. W. (2010). Analysis of electric field gradient
tensors at quadrupolar nuclei in common structural motifs. Concepts Magn. Reson., Part A.

[25] Kentgens A. P. M. (1997). A practical
guide to solid-state NMR of half-integer quadrupolar nuclei with some
applications to disordered systems. Geoderma.

[26] Ashbrook S. E. (2009). Recent
advances in solid-state NMR spectroscopy of quadrupolar nuclei. Phys. Chem. Chem. Phys..

[27] Berkson Z. J., Zhu R., Ehinger C., Lätsch L., Schmid S. P., Nater D., Pollitt S., Safonova O. V., Björgvinsdóttir S., Barnes A., Román-Leshkov Y., Price G. A., Sunley G. J., Copéret C. (2023). Active Site Descriptors from 95Mo
NMR Signatures of Silica-Supported Mo-Based Olefin Metathesis Catalysts. J. Am. Chem. Soc..

[28] Autschbach J., Zheng S. (2009). Chapter 1 Relativistic
Computations of NMR Parameters from First
Principles: Theory and Applications. Annu. Rep.
NMR Spectrosc..

[29] Autschbach J., Zheng S. (2008). Analyzing Pt chemical
shifts calculated from relativistic density
functional theory using localized orbitals: The role of Pt 5d lone
pairs. Magn. Reson. Chem..

[30] Autschbach J. (2014). Relativistic
calculations of magnetic resonance parameters: background and some
recent developments. Philos. Trans. R. Soc.,
A.

[31] O’Dell L. A., Schurko R. W., Harris K. J., Autschbach J., Ratcliffe C. I. (2011). Interaction Tensors and Local Dynamics in Common Structural
Motifs of Nitrogen: A Solid-State 14N NMR and DFT Study. J. Am. Chem. Soc..

[32] Staun S. L., Sergentu D.-C., Wu G., Autschbach J., Hayton T. W. (2019). Use of 15N NMR spectroscopy to probe
covalency in a
thorium nitride. Chem. Sci..

[33] Baker C. F., Seed J. A., Adams R. W., Lee D., Liddle S. T. (2023). 13Ccarbene
nuclear magnetic resonance chemical shift analysis confirms CeIV =
C double bonding in cerium­(iv)–diphosphonioalkylidene complexes. Chem. Sci..

[34] Du J., Hurd J., Seed J. A., Balász G., Scheer M., Adams R. W., Lee D., Liddle S. T. (2023). 31P Nuclear
Magnetic Resonance Spectroscopy as a Probe of Thorium–Phosphorus
Bond Covalency: Correlating Phosphorus Chemical Shift to Metal–Phosphorus
Bond Order. J. Am. Chem. Soc..

[35] Du J., Seed J. A., Berryman V. E. J., Kaltsoyannis N., Adams R. W., Lee D., Liddle S. T. (2021). Exceptional
uranium­(VI)-nitride
triple bond covalency from 15N nuclear magnetic resonance spectroscopy
and quantum chemical analysis. Nat. Commun..

[36] Holmes S. T., Schönzart J., Philips A. B., Kimball J. J., Termos S., Altenhof A. R., Xu Y., O’Keefe C. A., Autschbach J., Schurko R. W. (2024). Structure and bonding in rhodium
coordination compounds: a 103Rh solid-state NMR and relativistic DFT
study. Chem. Sci..

[37] Raynaud C., Norbert-Agaisse E., James B. R., Eisenstein O. (2020). 31P Chemical
Shifts in Ru­(II) Phosphine Complexes. A Computational Study of the
Influence of the Coordination Sphere. Inorg.
Chem..

[38] Gordon C. P., Lätsch L., Copéret C. (2021). Nuclear Magnetic
Resonance: A Spectroscopic
Probe to Understand the Electronic Structure and Reactivity of Molecules
and Materials. J. Phys. Chem. Lett..

[39] Hu J. Z., Alderman D. W., Ye C., Pugmire R., Grant D. (1993). An Isotropic
Chemical Shift-Chemical Shift Anisotropy Magic-Angle Slow-Spinning
2D NMR Experiment. J. Magn. Reson., Ser. A.

[40] Gan Z. (1992). High-resolution
chemical shift and chemical shift anisotropy correlation in solids
using slow magic angle spinning. J. Am. Chem.
Soc..

[41] Antzutkin O. N., Shekar S. C., Levitt M. H. (1995). Two-Dimensional Sideband Separation
in Magic-Angle-Spinning NMR. J. Magn. Reson.,
Ser. A.

[42] Dixon W. T. (1982). Spinning-sideband-free
and spinning-sideband-only NMR spectra in spinning samples. J. Chem. Phys..

[43] Baltisberger J. H., Walder B. J., Keeler E. G., Kaseman D. C., Sanders K. J., Grandinetti P. J. (2012). Communication:
Phase incremented echo train acquisition
in NMR spectroscopy. J. Chem. Phys..

[44] Lee D.-K., Wei Y., Ramamoorthy A. (2001). A Two-Dimensional Magic-Angle Decoupling and Magic-Angle
Turning Solid-State NMR Method: An Application to Study Chemical Shift
Tensors from Peptides That Are Nonselectively Labeled with 15N Isotope. J. Phys. Chem. B.

[45] Piveteau L., Ong T.-C., Walder B., Dirin D. N., Moscheni D., Schneider B., Bär J., Protescu L., Masciocchi N., Guagliardi A., Emsley L., Copéret C., Kovalenko M. V. (2018). Resolving the Core and the Surface of CdSe Quantum
Dots and Nanoplatelets Using Dynamic Nuclear Polarization Enhanced
PASS–PIETA NMR Spectroscopy. ACS Cent.
Sci..

[46] Chmelka, B. F. , Zwanziger, J. W. Solid-State NMR Line Narrowing Methods for Quadrupolar Nuclei: Double Rotation and Dynamic-Angle Spinning. In Solid-State NMR IV Methods and Applications of Solid-State NMR; Springer: 1994; pp 79–124.

[47] Dupree, R. Double Rotation NMR; Wiley-VCH: 2011.

[48] Medek A., Harwood J. S., Frydman L. (1995). Multiple-quantum magic-angle
spinning
NMR: A new method for the study of quadrupolar nuclei in solids. J. Am. Chem. Soc..

[49] Amoureux, J.-P. , Pruski, M. MQMAS NMR: Experimental Strategies and Applications; Wiley-VCH: 2008.

[50] Gan Z. (2019). Perspectives
on high-field and solid-state NMR methods of quadrupole nuclei. J. Magn. Reson..

[51] Pell A. J., Pintacuda G., Grey C. P. (2019). Paramagnetic NMR
in solution and
the solid state. Prog. Nucl. Magn. Reson. Spectrosc..

[52] Ashbrook S. E., Sneddon S. (2014). New Methods and Applications
in Solid-State NMR Spectroscopy
of Quadrupolar Nuclei. J. Am. Chem. Soc..

[53] Gan Z., Hung I., Wang X., Paulino J., Wu G., Litvak I. M., Gor’kov P. L., Brey W. W., Lendi P., Schiano J. L., Bird M. D., Dixon I. R., Toth J., Boebinger G. S., Cross T. A. (2017). NMR spectroscopy up to 35.2T using
a series-connected hybrid magnet. J. Magn. Reson..

[54] Bryce D. L. (2022). Chapter
One - Solid-state NMR of quadrupolar nuclei: Selected new methods
and applications. Annu. Rep. NMR Spectrosc..

[55] Xu J., Wang Q., Deng F. (2019). Metal Active
Sites and Their Catalytic
Functions in Zeolites: Insights from Solid-State NMR Spectroscopy. Acc. Chem. Res..

[56] Qi G., Wang Q., Xu J., Trébosc J., Lafon O., Wang C., Amoureux J.-P., Deng F. (2016). Synergic Effect
of Active Sites in Zinc-Modified ZSM-5 Zeolites as Revealed by High-Field
Solid-State NMR Spectroscopy. Angew. Chem.,
Int. Ed..

[57] Gao W., Wang Q., Qi G., Liang J., Wang C., Xu J., Deng F. (2023). Active Ensembles in Methane Dehydroaromatization over
Molybdenum/ZSM-5 Zeolite Identified by 2D 1H–95Mo Magic Angle
Spinning Nuclear Magnetic Resonance Correlation Spectroscopy. Angew. Chem., Int. Ed..

[58] Venkatesh A., Hanrahan M. P., Rossini A. J. (2017). Proton detection of MAS solid-state
NMR spectra of half-integer quadrupolar nuclei. Solid State Nucl. Magn. Reson..

[59] Rossini A. J., Hanrahan M. P., Thuo M. (2016). Rapid acquisition of
wideline MAS
solid-state NMR spectra with fast MAS, proton detection, and dipolar
HMQC pulse sequences. Phys. Chem. Chem. Phys..

[60] Venkatesh A., Ryan M. J., Biswas A., Boteju K. C., Sadow A. D., Rossini A. J. (2018). Enhancing
the Sensitivity of Solid-State NMR Experiments
with Very Low Gyromagnetic Ratio Nuclei with Fast Magic Angle Spinning
and Proton Detection. J. Phys. Chem. A.

[61] Lamahewage S. N. S., Atterbery B. A., Dorn R. W., Gi E., Kimball M. R., Blümel J., Vela J., Rossini A. J. (2024). Accelerated acquisition
of wideline solid-state NMR spectra of spin 3/2 nuclei by frequency-stepped
indirect detection experiments. Phys. Chem.
Chem. Phys..

[62] Atterberry B. A., Paluch P., Lamkins A. R., Huang W., Rossini A. J. (2025). Rapid Acquisition
of 103Rh Solid-State NMR Spectra by 31P Detection and Sideband Selective
Methods. J. Am. Chem. Soc..

[63] Kimball J. J., Schurko R. W. (2025). Acquisition of 1H-Detected
103Rh and 99Ru Solid-State
Nuclear Magnetic Resonance Spectra in Stationary Samples. J. Phys. Chem. Lett..

[64] Lätsch L., Kaul C. K., Yakimov A. V., Müller I. B., Hassan A., Perrone B., Aghazada S., Berkson Z. B., De Baerdemaeker T., Parvulescu A.-N., Seidel K., Teles J. H., Copéret C. (2023). NMR Signatures
and Electronic Structure of Ti Sites
in Titanosilicalite-1 from Solid-State 47/49Ti NMR Spectroscopy. J. Am. Chem. Soc..

[65] Berkson Z. J., Hsieh M.-F., Smeets S., Gajan D., Lund A., Lesage A., Xie D., Zones S. I., McCusker L. B., Baerlocher C., Chmelka B. F. (2019). Preferential Siting of Aluminum Heteroatoms
in the Zeolite Catalyst Al-SSZ-70. Angew. Chem.,
Int. Ed..

[66] Rossini A. J., Zagdoun A., Lelli M., Lesage A., Copéret C., Emsley L. (2013). Dynamic Nuclear Polarization Surface Enhanced NMR Spectroscopy. Acc. Chem. Res..

[67] Rossini A. J. (2018). Materials
Characterization by Dynamic Nuclear Polarization-Enhanced Solid-State
NMR Spectroscopy. J. Phys. Chem. Lett..

[68] Kobayashi T., Perras F. A., Slowing I. I., Sadow A. D., Pruski M. (2015). Dynamic Nuclear
Polarization Solid-State NMR in Heterogeneous Catalysis Research. ACS Catal..

[69] Ni Q. Z., Daviso E., Can T. V., Markhasin E., Jawla S. K., Swager T. M., Temkin R. J., Herzfeld J., Griffin R. G. (2013). High Frequency Dynamic Nuclear Polarization. Acc. Chem. Res..

[70] Moroz I. B., Leskes M. (2022). Dynamic Nuclear Polarization
Solid-State NMR Spectroscopy
for Materials Research. Annu. Rev. Mater. Res..

[71] Rankin A. G. M., Trébosc J., Pourpoint P., Amoureux J.-P., Lafon O. (2019). Recent developments in MAS DNP-NMR
of materials. Solid State Nucl. Magn. Reson..

[72] Hall D. A., Maus D. C., Gerfen G. J., Inati S. J., Becerra L. R., Dahlquist F. W., Griffin R. G. (1997). Polarization-Enhanced NMR Spectroscopy
of Biomolecules in Frozen Solution. Science.

[73] Schurko R. W. (2013). Ultra-Wideline
Solid-State NMR Spectroscopy. Acc. Chem. Res..

[74] Koppe J., Bußkamp M., Hansen M. R. (2021). Frequency-Swept Ultra-Wideline Magic-Angle
Spinning NMR Spectroscopy. J. Phys. Chem. A.

[75] Koppe J., Frerichs J. E., Hansen M. R. (2023). Pushing the Detection Limit of Static
Wideline NMR Spectroscopy Using Ultrafast Frequency-Swept Pulses. J. Phys. Chem. Lett..

[76] Larsen F. H., Jakobsen H. J., Ellis P. D., Nielsen N. C. (1997). Sensitivity-Enhanced
Quadrupolar-Echo NMR of Half-Integer Quadrupolar Nuclei. Magnitudes
and Relative Orientation of Chemical Shielding and Quadrupolar Coupling
Tensors. J. Phys. Chem. A.

[77] Lipton A. S., Sears J. A., Ellis P. D. (2001). A General
Strategy for the NMR Observation
of Half-Integer Quadrupolar Nuclei in Dilute Environments. J. Magn. Reson..

[78] Yakimov A. V., Ravi M., Verel R., Sushkevich S. L., van Bokhoven J. A., Copéret C. (2022). Structure
and Framework Association
of Lewis Acid Sites in MOR Zeolite. J. Am. Chem.
Soc..

[79] Thurber K. R., Tycko R. (2009). Measurement of sample temperatures under magic-angle spinning from
the chemical shift and spin-lattice relaxation rate of 79Br in KBr
powder. J. Magn. Reson..

[80] Bielecki A., Burum D. P. (1995). Temperature Dependence
of 207Pb MAS Spectra of Solid
Lead Nitrate. An Accurate, Sensitive Thermometer for Variable-Temperature
MAS. J. Magn. Reson., Ser. A.

[81] Smith M. E. (2021). Recent
progress in solid-state nuclear magnetic resonance of half-integer
spin low-γ quadrupolar nuclei applied to inorganic materials. Magn. Reson. Chem..

[82] Ramsey N. F. (1950). Magnetic
Shielding of Nuclei in Molecules. Phys. Rev..

[83] van
Lenthe E., Ehlers A., Baerends E.-J. (1999). Geometry optimizations
in the zero order regular approximation for relativistic effects. J. Chem. Phys..

[84] Lenthe E. V., Baerends E. J., Snijders J. G. (1993). Relativistic
regular two-component
Hamiltonians. J. Chem. Phys..

[85] van
Lenthe E., Baerends E. J., Snijders J. G. (1994). Relativistic total
energy using regular approximations. J. Chem.
Phys..

[86] van
Lenthe E., Snijders J. G., Baerends E. J. (1996). The zero-order regular
approximation for relativistic effects: The effect of spin-orbit coupling
in closed shell molecules. J. Chem. Phys..

[87] van
Lenthe E., van Leeuwen R., Baerends E. J., Snijders J. G. (1996). Relativistic
regular two-component Hamiltonians. Int. J.
Quantum Chem..

[88] Pigliapochi R., Pell A. J., Seymour I. D., Grey C. P., Ceresoli D., Kaupp M. (2017). DFT investigation of
the effect of spin-orbit coupling on the NMR
shifts in paramagnetic solids. Phys. Rev. B.

[89] Copéret C., Berkson Z., Chan K.-W., de Jesus Silva J., Gordon C. P., Pucino M., Zhizhko P. (2021). Olefin metathesis:
what have we learned about homogeneous and heterogeneous catalysts
from surface organometallic chemistry?. Chem.
Sci..

[90] Hoveyda A. H., Zhugralin A. R. (2007). The remarkable metal-catalysed olefin metathesis reaction. Nature.

[91] Schrock R. R., Hoveyda A. H. (2003). Molybdenum and Tungsten
Imido Alkylidene Complexes
as Efficient Olefin-Metathesis Catalysts. Angew.
Chem., Int. Ed..

[92] Ehrhorn H., Tamm M. (2019). Well-Defined Alkyne
Metathesis Catalysts: Developments and Recent
Applications. Chem. - Eur. J..

[93] Fürstner A. (2021). The Ascent
of Alkyne Metathesis to Strategy-Level Status. J. Am. Chem. Soc..

[94] Koh M.-J., Nguyen T. T., Lam J. K., Torker S., Hyvl J., Schrock R. R., Hoveyda A. H. (2017). Molybdenum
chloride catalysts for
Z-selective olefin metathesis reactions. Nature.

[95] Fürstner A. (2000). Olefin Metathesis
and Beyond. Angew. Chem., Int. Ed..

[96] Fürstner A. (2013). Alkyne Metathesis
on the Rise. Angew. Chem., Int. Ed..

[97] Estes D. P., Gordon C. P., Fedorov A., Liao W.-C., Ehrhorn E., Bittner C., Zier M. L., Bockfeld D., Chan K. W., Eisenstein O., Raynaud C., Tamm M., Copéret C. (2017). Molecular
and Silica-Supported Molybdenum Alkyne Metathesis Catalysts: Influence
of Electronics and Dynamics on Activity Revealed by Kinetics, Solid-State
NMR, and Chemical Shift Analysis. J. Am. Chem.
Soc..

[98] Hillenbrand J., Leutzsch M., Yiannakas E., Gordon C. P., Wille C., Nöthling N., Copéret C., Fürstner A. (2020). “Canopy
Catalysts” for Alkyne Metathesis: Molybdenum Alkylidyne Complexes
with a Tripodal Ligand Framework. J. Am. Chem.
Soc..

[99] Minelli M., Enemark J. H., Brownlee R. T. C., O’connor M. J., Wedd M. J. (1985). The nuclear magnetic resonance properties of chromium,
molybdenum and tungsten compounds. Coord. Chem.
Rev..

[100] Knight C. T. G., Turner G. L., Kirkpatrick R. J., Oldfield E. (1986). Solid-state tungsten-183
nuclear magnetic resonance
spectroscopy. J. Am. Chem. Soc..

[101] Merwin L. H., Sebald A. (1992). Cross-polarisation to low-γ
nuclei: the first 183W CPMAS spectra. Solid
State Nucl. Magn. Reson..

[102] Haouas M., Trébosc J., Roch-Marchal C., Cadot E., Taulelle F., Martineau-Corcos C. (2017). High-field
95Mo and 183W static and MAS NMR study of polyoxometalates. Magn. Reson. Chem..

[103] Berkson Z. J., Bernhardt M., Copéret C. (2024). Solid-State
183W NMR Spectroscopy as a High-Resolution Probe of Polyoxotungstate
Structures and Dynamics. J. Phys. Chem. Lett..

[104] Poater A., Solans-Monfort X., Clot E., Copéret C., Eiesnstein O. (2007). Understanding d0-Olefin Metathesis Catalysts: Which
Metal, Which Ligands?. J. Am. Chem. Soc..

[105] Amakawa K., Wrabetz S., Kröhnert J., Tzolova-Müller G., Schlögl R., Trunschke A. (2012). In Situ Generation of Active Sites in Olefin Metathesis. J. Am. Chem. Soc..

[106] Gani T. Z. H., Berkson Z. J., Zhu R., Kang J. H., Di Iorio J. R., Chan K. W., Consoli D. F., Shaikh S. K., Copéret C., Román-Leshkov Y. (2023). Promoting
active site
renewal in heterogeneous olefin metathesis catalysts. Nature.

[107] Chan K. W., Mance D., Safonova O. V., Copéret C. (2019). Well-Defined
Silica-Supported Tungsten­(IV)–Oxo Complex: Olefin Metathesis
Activity, Initiation, and Role of Bro̷nsted Acid Sites. J. Am. Chem. Soc..

[108] Mougel V., Chan K. W., Siddiqi G., Kawakita K., Nagae H., Tsurugi H., Mashima K., Safonova O., Copéret C. (2016). Low Temperature Activation of Supported
Metathesis
Catalysts by Organosilicon Reducing Agents. ACS Cent. Sci..

[109] Amakawa K., Sun L., Guo C., Hävecker M., Kube P., Wachs I. E., Lwin S., Frenkel A. I., Patlolla A., Hermann K., Schlögl R., Trunschke A. (2013). How Strain Affects the Reactivity of Surface Metal
Oxide Catalysts. Angew. Chem., Int. Ed..

[110] Berkson Z. J., Bernhardt M., Schlapansky S. L., Benedikter M. J., Buchmeiser M. R., Price G. A., Sunley G. J., Copéret C. (2022). Olefin-Surface
Interactions: A Key Activity Parameter
in Silica-Supported Olefin Metathesis Catalysts. JACS Au.

[111] Gao W., Qi G., Wang Q., Wang W., Li S., Hung I., Gan Z., Xu J., Deng F. (2021). Dual Active
Sites on Molybdenum/ZSM-5 Catalyst for Methane Dehydroaromatization:
Insights from Solid-State NMR Spectroscopy. Angew. Chem., Int. Ed..

[112] Berkson Z. J., Björgvinsdóttir S., Yakimov A., Gioffrè D., Korzyński M. D., Barnes A. B., Copéret C. (2022). Solid-State NMR Spectra of Protons
and Quadrupolar Nuclei at 28.2 T: Resolving Signatures of Surface
Sites with Fast Magic Angle Spinning. JACS Au.

[113] Zheng H., Ma D., Bao X., Hu J. Z., Kwak J. H., Wang Y., Peden C. H. F. (2008). Direct Observation
of the Active Center for Methane Dehydroaromatization Using an Ultrahigh
Field 95Mo NMR Spectroscopy. J. Am. Chem. Soc..

[114] Nater D.
F., Kaul C. J., Lätsch L., Tsurugi H., Mashima K., Copéret C. (2022). Olefin Metathesis
Catalysts Generated In Situ from Molybdenum­(VI)-Oxo Complexes by Tuning
Pendant Ligands. Chem. - Eur. J..

[115] Kaiser S. K., Chen Z., Faust Aki D., Mitchell S., Pérez-Ramírez J. (2020). Single-Atom
Catalysts across the Periodic Table. Chem. Rev..

[116] Priqueler J. R. L., Butler I. S., Rochon F. D. (2006). An Overview
of 195Pt
Nuclear Magnetic Resonance Spectroscopy. Appl.
Spectrosc. Rev..

[117] Pregosin P. S. (1986). Platinum NMR Spectroscopy. Annu.
Rep. NMR Spectrosc..

[118] Venkatesh A., Gioffrè D., Atterberry B. A., Rochlitz L., Carnahan S. L., Wang Z., Menzildjian G., Lesage A., Copéret C., Rossini A. J. (2022). Molecular and Electronic
Structure of Isolated Platinum Sites Enabled by the Expedient Measurement
of 195Pt Chemical Shift Anisotropy. J. Am. Chem.
Soc..

[119] Gilbert T. M., Ziegler T. (1999). Prediction of 195Pt NMR Chemical
Shifts by Density Functional Theory Computations: The Importance of
Magnetic Coupling and Relativistic Effects in Explaining Trends. J. Phys. Chem. A.

[120] Ishizaka Y., Arai N., Matsumoto K., Nagashima H., Takeuchi K., Fukaya N., Yasuda H., Sato K., Choi J.-C. (2021). Bidentate Disilicate Framework for
Bis-Grafted Surface Species. Chem. - Eur. J..

[121] Camacho-Bunquin J., Ferrandon M., Sohn H., Yang D., Liu C., Ignacio-de
Leon P. A., Perras F. A., Pruski M., Stair P. C., Delferro M. (2018). Chemoselective Hydrogenation with
Supported Organoplatinum­(IV) Catalyst on Zn­(II)-Modified Silica. J. Am. Chem. Soc..

[122] Harris K. J., Lupulescu A., Lucier B. E. G., Frydman L., Schurko R. W. (2012). Broadband adiabatic
inversion pulses for cross polarization
in wideline solid-state NMR spectroscopy. J.
Magn. Reson..

[123] MacGregor A. W., O’Dell L. A., Schurko R. W. (2011). New methods for
the acquisition of ultra-wideline solid-state NMR spectra of spin-1/2
nuclides. J. Magn. Reson..

[124] O’Dell L. A., Schurko R. W. (2008). QCPMG using adiabatic
pulses for
faster acquisition of ultra-wideline NMR spectra. Chem. Phys. Lett..

[125] Wang Z., Robinson T. C., Gioffrè D., Lukas R., Gajan D., Rossini A. J., Copéret C., Lesage A. (2024). Natural abundance 195Pt-13C correlation NMR spectroscopy
on surfaces enabled by fast MAS dynamic nuclear polarization. J. Magn. Reson. Open.

[126] Bayzou R., Trébosc J., Hung I., Gan Z., Lafon O., Amoureux J. P. (2022). Indirect
NMR detection via proton
of nuclei subject to large anisotropic interactions, such as 14N,
195Pt, and 35Cl, using the T-HMQC sequence. J. Chem. Phys..

[127] Atterberry B. A., Wimmer E., Estes D. P., Rossini A. J. (2023). Acceleration
of indirect detection 195Pt solid-state NMR experiments by sideband
selective excitation or alternative indirect sampling schemes. J. Magn. Reson..

[128] Jaroszewicz M. J., Altenhof A. R., Schurko R. W., Frydman L. (2021). Sensitivity
Enhancement by Progressive Saturation of the Proton Reservoir: A Solid-State
NMR Analogue of Chemical Exchange Saturation Transfer. J. Am. Chem. Soc..

[129] Wolf T., Goobes Y., Frydman L. (2024). Sensitivity
Enhancement
of Ultra-Wideline NMR by Progressive Saturation of the Proton Reservoir
Under Magic-Angle Spinning. Chemphyschem.

[130] Lucier B. E. G., Reidel A. R., Schurko R. W. (2011). Multinuclear
solid-state
NMR of square-planar platinum complexes - Cisplatin and related systems. Can. J. Chem..

[131] Kobayashi T., Perras F. A., Goh T. W., Metz T. L., Huang W., Pruski M. (2016). DNP-Enhanced Ultrawideline
Solid-State
NMR Spectroscopy: Studies of Platinum in Metal–Organic Frameworks. J. Phys. Chem. Lett..

[132] Venkatesh A., Lund A., Rochlitz L., Jabbour R., Gordon C. P., Menzildjian G., Viger-Gravel J., Berruyer P., Gajan D., Copéret C., Lesage A., Emsley L. (2020). The Structure of Molecular and Surface
Platinum Sites Determined by DNP-SENS and Fast MAS 195Pt Solid-State
NMR Spectroscopy. J. Am. Chem. Soc..

[133] Wang Z., Völker L. A., Robinson T. C., Kaeffer N., Menzildjian G., Jabbour R., Venkatesh A., Gajan D., Rossini A. J., Copéret C., Lesage A. (2022). Speciation and Structures in Pt Surface
Sites Stabilized
by N-Heterocyclic Carbene Ligands Revealed by Dynamic Nuclear Polarization
Enhanced Indirectly Detected 195Pt NMR Spectroscopic Signatures and
Fingerprint Analysis. J. Am. Chem. Soc..

[134] Atterberry B. A., Wimmer E. J., Klostermann S., Frey W., Kästner J., Estes D. P., Rossini A. J. (2025). Structural
characterization of surface immobilized platinum hydrides by sensitivity-enhanced
195Pt solid state NMR spectroscopy and DFT calculations. Chem. Sci..

[135] McCullough K. E., Pezcak I. L., Kennedy R. M., Wang Y.-Y., Lin J., Wu X., Paterson A. L., Perras F. A., Hall J., Kropf A. J., Hackler R. A., Shin Y., Niklas J., Poluektov O. G., Wen J., Huang W., Sadow A. D., Poeppelmeier K. R., Delferro M., Ferrandon M. S. (2023). Synthesis
of platinum nanoparticles on strontium titanate nanocuboids via surface
organometallic grafting for the catalytic hydrogenolysis of plastic
waste. J. Mater. Chem. A.

[136] Hansen C., Docherty S. R., Cao W., Yakimov A. V., Copéret C. (2024). 109Ag NMR chemical shift as a descriptor for Bro̷nsted
acidity from molecules to materials. Chem. Sci..

[137] Amanullah S., Cao W., Brack E., Plodinec M., Copéret C. (2024). Surface Coordination
Chemistry of Graphitic Carbon
Nitride from Ag Molecular Probes. Angew. Chem.,
Int. Ed..

[138] Koppe J., Yakimov A. V., Gioffrè D., Usteri M.-E., Vosegaard T., Pintacuda G., Lesage A., Pell A. J., Mitchell S., Pérez-Ramírez J., Copéret C. (2025). Coordination environments of platinum single atom catalysts
from NMR signatures. Nature.

[139] Mustarelli P., Tomasi C., Quartarone E., Magistris A., Cutroni M., Mandanici A. (1998). Structure
and cation dynamics in the system AgI: Ag2MoO4: A 109Ag NMR study. Phys. Rev. B.

[140] Rakhmatullin A., Brusko V. V., Shcherbitskaya E. R., Polovov I. B., Bakirov R., Bessada C. (2022). Solid-state 31P and
109Ag CP/MAS NMR as a powerful tool for studying of silver­(I) complexes
with N-thiophosphorylated thiourea and thioamide ligands. Magn. Reson. Chem..

[141] Looser H., Brinkmann D. (1985). 109Ag chemical
shifts in some solid
compounds. J. Magn. Reson..

[142] Lesage A., Lelli M., Gajan D., Caporini M. A., Vitzthum V., Miéville P., Alauzun P., Roussey A., Thieuleux C., Mehdi A., Bodenhausen J., Copéret C., Emsley L. (2010). Surface Enhanced NMR Spectroscopy
by Dynamic Nuclear Polarization. J. Am. Chem.
Soc..

[143] Liao W.-C., Ghaffari B., Gordon C. P., Xu J., Copéret C. (2018). Dynamic Nuclear
Polarization Surface Enhanced NMR spectroscopy
(DNP SENS): Principles, protocols, and practice. Curr. Opin. Colloid Interface Sci..

[144] Pavlovskaya G. E., Horton-Garcia C. F., Dybowki C., Corbin D. R., Meersmann T. (2004). Metallic Clusters
and Color Changes in Silver-Exchanged
Zeolites: 109Ag Solid State NMR and Optical Studies. J. Phys. Chem. B.

[145] Ren J., Eckert H. (2013). Anion Distribution in Superionic Ag3PO4–AgI
Glasses Revealed by Dipolar Solid-State NMR. J. Phys. Chem. C.

[146] Hamaed H., Lo A. Y. H., May L. J., Taylor J. M., Shimizu J. H., Schurko R. W. (2008). Investigation of
Silver-Containing
Layered Materials and Their Interactions with Primary Amines Using
Solid-State 109Ag and 15N NMR Spectroscopy and First Principles Calculations. Inorg. Chem..

[147] Blais-Roberge M., Santagneli S. H., Messaddeq S. H., Rioux M., Ledemi Y., Eckert H., Messaddeq Y. (2017). Structural
Characterization of AgI–AgPO3–Ag2WO4 Superionic Conducting
Glasses by Advanced Solid-State NMR Techniques. J. Phys. Chem. C.

[148] Lumata L., Merritt M. E., Hashami Z., Ratnakar S. J., Kovacs Z. (2012). Production and NMR Characterization
of Hyperpolarized
107,109Ag Complexes. Angew. Chem., Int. Ed..

[149] Chen F., Wasylishen R. E. (2010). Structural characterization of silver
dialkylphosphite salts using solid-state 109Ag and 31P NMR spectroscopy,
IR spectroscopy and DFT calculations. Magn.
Reson. Chem..

[150] Claverie J. P., Schaper F. (2013). Ziegler-Natta catalysis: 50 years
after the Nobel Prize. MRS Bull..

[151] Kashiwa N. (2004). The discovery and progress of MgCl2-supported
TiCl4
catalysts. J. Polym. Sci., Part A: Polym. Chem..

[152] Kumawat J., Gupta V. K. (2020). Fundamental aspects of heterogeneous
Ziegler–Natta olefin polymerization catalysis: an experimental
and computational overview. Polym. Chem..

[153] Groppo E., Seenivasan K., Barzan C. (2013). The potential of spectroscopic
methods applied to heterogeneous catalysts for olefin polymerization. Catal. Sci. Technol..

[154] Piovano A., Groppo E. (2022). Flexible ligands in
heterogeneous
catalysts for olefin polymerization: Insights from spectroscopy. Coord. Chem. Rev..

[155] Ashuiev A., Humbert M., Norsic S., Blahut J., Gajan D., Searles K., Klose D., Lesage A., Pintacuda G., Raynaud J., Monteil V., Copéret C., Jeschke G. (2021). Spectroscopic Signature and Structure of the Active
Sites in Ziegler–Natta Polymerization Catalysts Revealed by
Electron Paramagnetic Resonance. J. Am. Chem.
Soc..

[156] Vittadello M., Stallworth P. E., Alagmir F. M., Suarez S., Abbrent S., Drain C. M., Di Noto V., Greenbaum S. G. (2006). Polymeric
δ-MgCl2 nanoribbons. Inorg. Chim. Acta.

[157] Sabisch S., Kakiuchi Y., Docherty S. R., Yakimov A. V., Copéret C. (2023). Geometry and Local Environment of
Surface Sites in
Vanadium-Based Ziegler–Natta Catalysts from 51V Solid-State
NMR Spectroscopy. J. Am. Chem. Soc..

[158] Credendino R., Busico V., Causà M., Barone V., Budzelaar P. H. M., Zicovich-Wilson C. (2009). Periodic DFT
modeling of bulk and surface properties of MgCl2. Phys. Chem. Chem. Phys..

[159] D’Amore M., Credendino R., Budzelaar P. H. M., Causà M., Busico V. (2012). A periodic hybrid DFT
approach (including
dispersion) to MgCl2-supported Ziegler–Natta catalysts - 1:
TiCl4 adsorption on MgCl2 crystal surfaces. J. Catal..

[160] Humbert M., Norsic S., Raynaud J., Monteil V. (2019). Activity Enhancement
of MgCl2-supported Ziegler-Natta Catalysts by Lewis-acid Pre-treatment
for Ethylene Polymerization. Chin. J. Polym.
Sci..

[161] Yakimov A. V., Kaul C. J., Kakiuchi Y., Sabisch S., Bolner F. M., Raynaud J., Monteil V., Berruyer P., Copéret C. (2024). Well-Defined Ti Surface Sites in Ziegler–Natta
Pre-Catalysts from 47/49Ti Solid-State Nuclear Magnetic Resonance
Spectroscopy. J. Phys. Chem. Lett..

[162] Corma A., Martinez A. (1995). Zeolites and Zeotypes as catalysts. Adv. Mater..

[163] Nemeth L., Bare S. R. (2014). Chapter One - Science and Technology
of Framework Metal-Containing Zeotype Catalysts. Adv. Catal..

[164] Xu H., Wu P. (2017). Recent Progresses in
Titanosilicates. Chin. J. Chem..

[165] Taramasso, M. ; Perego, G. ; Notari, B. Preparation of porous crystalline synthetic material comprised of silicon and titanium oxides. US4410501A, 1983.

[166] Smeets V., Gaigneaux E. M., Debecker D. P. (2022). Titanosilicate Epoxidation
Catalysts: A Review of Challenges and Opportunities. ChemCatChem.

[167] Guo Q., Sun K., Feng Z., Li G., Guo M., Fan F., Li C. (2012). A Thorough Investigation of the Active Titanium Species
in TS-1 Zeolite by In Situ UV Resonance Raman Spectroscopy. Chem. - Eur. J..

[168] Wang J., Chen Z., Yu Y., Tang Z., Shen K., Wang R., Liu H., Huang X., Liu Y. (2019). Hollow core–shell structured
TS-1@S-1 as an efficient catalyst
for alkene epoxidation. RSC Adv..

[169] Hassan A., Quinn C. M., Struppe J., Sergeyev I. V., Zhang C., Guo C., Runge B., Thient T., Dao H. H., Jaroniec C. P., Berbon M., Lends A., Habenstein B., Loquet A., Kuemmerle R., Perrone B., Gronenborn A. M., Polenova T. (2020). Sensitivity boosts
by the CPMAS CryoProbe for challenging biological assemblies. J. Magn. Reson..

[170] d’Espinose de Lacaillerie J.-B., Fretigny C., Massiot D. (2008). MAS NMR spectra
of quadrupolar nuclei in disordered solids: The Czjzek model. J. Magn. Reson..

[171] Spanjers C. S., Guillo P., Tilley T. D., Janik M. J., Rioux R. M. (2017). Identification of Second Shell Coordination
in Transition
Metal Species Using Theoretical XANES: Example of Ti–O–(C,
Si, Ge) Complexes. J. Phys. Chem. A.

[172] Schirò A., Carlon A., Parigi G., Murshudov G., Calderone V., Ravera E., Luchinat C. (2020). On the complementarity
of X-ray and NMR data. J. Struct. Biol.: X.

[173] Tycko R. (2013). NMR at Low and Ultralow Temperatures. Acc.
Chem. Res..

[174] Blanc F., Basset J.-M., Copéret C., Sinha A., Tonzetich Z. J., Schrock R. R., Solans-Monfort X., Clot E., Eisenstein O., Lesage A., Emsley L. (2008). Dynamics of
Silica-Supported Catalysts Determined by Combining Solid-State NMR
Spectroscopy and DFT Calculations. J. Am. Chem.
Soc..

[175] Cordova M., Moutzouri P., Nilsson Lill S. O., Cousen A., Kearns M., Norberg S. T., Svensk
Ankarberg A., McCabe J., Pinon A. C., Schantz S., Emsley L. (2023). Atomic-level structure determination of amorphous molecular
solids by NMR. Nat. Commun..

[176] Salager E., Day G. M., Stein R. S., Pickard C. J., Elena B., Emsley L. (2010). Powder Crystallography by Combined
Crystal Structure Prediction and High-Resolution 1H Solid-State NMR
Spectroscopy. J. Am. Chem. Soc..

[177] Emsley L. (2025). Spiers Memorial Lecture: NMR crystallography. Faraday Discuss..

[178] Martineau, C. ; Senker, J. ; Taulelle, F. Chapter One - NMR Crystallography. In Annual Reports on NMR Spectroscopy; Academic Press: 2014, pp 1–57.

